# A Review of Multiparameter Fiber-Optic Distributed Sensing Techniques for Simultaneous Measurement of Temperature, Strain, and Environmental Effects

**DOI:** 10.3390/s25237225

**Published:** 2025-11-26

**Authors:** Artem Turov, Andrei Fotiadi, Dmitry Korobko, Ivan Panyaev, Maxim Belokrylov, Fedor Barkov, Yuri Konstantinov, Dmitriy Kambur, Airat Sakhabutdinov, Mohammed Qaid

**Affiliations:** 1 Institute of Continuous Media Mechanics of the Ural Branch of the Russian Academy of Sciences, Academician Korolev St. 1, Perm 614013, Russia; artemtur442@gmail.com (A.T.); belokrylovme@gmail.com (M.B.); barkov.f@permsc.ru (F.B.); yuri.al.konstantinov@ro.ru (Y.K.); kambur.dima@gmail.com (D.K.); 2 General Physics Department, Applied Mathematics and Mechanics Faculty, Perm National Research Polytechnic University, Komsomolsky Prospect, 29, Perm 614990, Russia; 3 S.P. Kapitsa Research Institute of Technology, Ulyanovsk State University, 42 Leo Tolstoy Street, Ulyanovsk 432970, Russia; korobkotam@rambler.ru (D.K.); panyaev.ivan@rambler.ru (I.P.); 4 Electromagnetism and Telecommunication Department, University of Mons, B-7000 Mons, Belgium; 5 Department of Radiophotonics and Microwave Technologies, Kazan National Research Technical University Named After A. N. Tupolev—KAI, 10 K.Marx St., Kazan 420111, Russia; azhsakhabutdinov@kai.ru (A.S.); kaidmr@stud.kai.ru (M.Q.)

**Keywords:** distributed fiber-optic sensors, multiparameter sensing, coherent optical frequency-domain reflectometry (C-OFDR), low-coherence optical time-domain reflectometry (LC-OTDR), coherent phase-sensitive time-domain reflectometry (Φ-OTDR), Brillouin optical time-domain reflectometry (BOTDR), temperature and strain discrimination, cross-sensitivity compensation

## Abstract

This review summarizes recent progress and emerging trends in multiparameter optical fiber sensing, emphasizing techniques that enable the simultaneous measurement of temperature, strain, acoustic waves, pressure, and other environmental quantities within a single sensing network. Such capabilities are increasingly important for structural health monitoring, environmental surveillance, industrial diagnostics, and geophysical observation, where multiple stimuli act on the fiber simultaneously. The paper outlines the physical principles and architectures underlying these systems and focuses on strategies for compensating and decoupling cross-sensitivity among measured parameters. Special attention is devoted to advanced distributed sensing schemes based on coherent optical frequency-domain reflectometry (C-OFDR), coherent phase-sensitive time-domain reflectometry (Φ-OTDR), and Brillouin optical time-domain reflectometry (BOTDR). Their theoretical foundations, their signal-processing algorithms, and the design modifications that improve parameter discrimination and accuracy are analyzed and compared. The review also highlights the roles of polarization and mode diversity and the growing application of machine-learning techniques in the interpretation and calibration of data. Finally, current challenges and promising directions for the next generation of fiber-optic multiparameter sensors are outlined, with a view toward high-resolution, low-cost, and field-deployable solutions for real-world monitoring applications.

## 1. Introduction

Distributed fiber-optic sensors (DFOS) represent one of the most accurate and versatile means of measuring physical quantities in real-world settings [1,2,3]. These systems are extensively employed across aerospace, automotive, civil, medical, and chemical industries. Their core strength lies in the simultaneous monitoring of strain, pressure, and temperature in solid structures, fluids, and composite materials [4,5,6,7]. They also play a vital role in environmental monitoring, enabling the detection of pollutants in air, water, and soil, as well as geophysical phenomena such as microseismic events or potential earthquake precursors [8,9,10,11]. In security and safety systems, fiber-optic sensors are used for the early detection of hazardous events—such as gas leaks, smoke, or fire—while simultaneously monitoring the structural integrity of bridges, tunnels, pipelines, and industrial equipment [12,13]. Furthermore, in geophysical exploration, fiber-based systems support subsurface imaging, fault-line monitoring, and mineral resource assessment [14,15].

In comparison with conventional sensing technologies, fiber-optic sensors offer numerous advantages, including high precision, low noise, long-term stability, and durability. Their immunity to electromagnetic interference and corrosion, combined with their ease of deployment and minimal calibration requirements, make them ideal for large-scale and long-term monitoring in both remote and urban environments.

However, the very property that enables optical fibers to sense multiple physical quantities simultaneously also introduces measurement ambiguity due to cross-sensitivity [16,17]. Many sensing mechanisms—whether based on Rayleigh scattering, Brillouin interactions, or refractive index modulation—respond simultaneously to several external influences, such as temperature and strain. Consequently, it becomes difficult to isolate the effect of a single parameter without interference from others. This ambiguity can significantly degrade the accuracy of measurement and lead to misinterpretation of sensor data unless compensated through proper system design or signal processing.

To overcome these challenges, extensive research has been devoted to the decoupling and compensation of cross-sensitivity effects. This effort encompasses several strategies:

(1) Hybrid sensor architectures that combine different scattering mechanisms (Rayleigh, Brillouin, Raman) to exploit their distinct sensitivities;
(2) Fiber designs engineered for selective or differential responses to specific parameters;
(3) Advanced interrogation and signal-processing techniques, which employ spectral decomposition, correlation analysis, or model-based demodulation to separate overlapping contributions.

Distributed reflectometry techniques, such as Coherent Optical Frequency-Domain Reflectometry (C-OFDR) [18], Coherent Phase-Sensitive Optical Time-Domain Reflectometry (Φ-OTDR) [19], and Brillouin Optical Time-Domain Reflectometry (BOTDR) [20], have become key tools in this context, enabling spatially resolved parameter mapping along the fiber length with high sensitivity and a wide range. More recently, data-driven and machine-learning approaches have emerged as powerful complements to analytical models. These algorithms can extract hidden patterns and nonlinear correlations from complex spectral or temporal datasets, enabling accurate estimation of multiple parameters even when theoretical calibration is difficult or incomplete.

This review provides a comprehensive overview of the current state of multiparameter optical fiber sensing, focusing on technologies that achieve the decoupling of temperature, strain, and other environmental effects in distributed configurations based on Rayleigh and Brillouin scattering. It discusses the underlying physical principles, the mathematical models describing each sensing mechanism, and the sensor architectures and interrogation strategies used for the simultaneous measurement of multiple quantities. Special attention is given to recent developments in polarization-maintaining fibers, multicore and few-mode fibers, and hybrid configurations that integrate multiple sensing modalities within a single platform. By critically analyzing the capabilities, limitations, and future trends in fiber-optic multiparameter sensing, this paper aims to serve as a comprehensive reference for researchers and engineers engaged in developing and deploying next-generation distributed fiber-optic sensor systems in scientific, industrial, and environmental applications.

The remainder of this paper is organized as follows. Section 2 introduces the fundamental concepts of cross-sensitivity and linearization in multiparameter fiber-optic sensing, highlighting mathematical frameworks for parameter discrimination and accuracy estimation. Section 3 reviews recent progress in coherent phase-sensitive reflectometry (Φ-OTDR) and related distributed techniques, summarizing approaches to simultaneous strain, temperature, and acoustic measurement. Section 4 focuses on coherent optical frequency-domain reflectometry (C-OFDR), examining discrimination algorithms, correlation-based demodulation, and novel hybrid configurations. Finally, Section 5 outlines emerging research directions, including polarization- and mode-diversity strategies, integration with machine learning, and the development of compact, field-deployable systems for advanced environmental and structural monitoring.

## 2. Approaches to Solving the Problem of Cross-Sensitivity

As noted in the Introduction, the parameters measured by fiber-optic sensors are usually affected by several external factors simultaneously. For example, both temperature and mechanical deformation alter the refractive index and length of the optical fiber. When only one measurable quantity is available, it is impossible to determine which effect caused the observed change. In such cases, multiparameter measurements can be used: the sensor registers several independent parameters that are subsequently converted into the desired physical quantities. This conversion can be carried out either by an analytical solution of a system of equations or using machine-learning techniques that learn the mapping between measured and sought quantities.

Consider a simplified case of a sensor that registers two measurable quantities, $P_{1}$ and $P_{2}$, each depending on two physical influences—temperature T and strain σ. The functional dependencies can be expressed as a system of equations:

(1) $$\left\{\begin{array}{c} P_{1}=f_{1}(T,\sigma )\\ P_{2}=f_{2}(T,\sigma ) \end{array}\right\}.$$

In the general case, system (1) can have several solutions with respect to *T* and *σ*, which makes it impossible to unambiguously determine the physical quantities. Therefore, in practice, systems with linear or quasi-linear responses are preferred. Since any smooth function of two variables can be represented by a Taylor expansion, we can linearize the dependences of Equation (1) near a working point $\left(T_{0},\sigma_{0}\right)$ as follows:

(2) $$\begin{array}{l} f(T,\sigma )=f(T_{0},\sigma_{0})+\frac{\partial f(T_{0},\sigma_{0})}{\partial T}(T-T_{0})+\frac{\partial f(T_{0},\sigma_{0})}{\partial \sigma }(\sigma -\sigma_{0})+\\ +\frac{1}{2}\frac{\partial^{2}f(T_{0},\sigma_{0})}{\partial T^{2}}{\left(T-T_{0}\right)}^{2}+\frac{1}{2}\frac{\partial^{2}f(T_{0},\sigma_{0})}{\partial \sigma^{2}}{\left(\sigma -\sigma_{0}\right)}^{2}+\frac{\partial^{2}f(T_{0},\sigma_{0})}{\partial T\partial \sigma }(T-T_{0})(\sigma -\sigma_{0})+\ldots \end{array}$$

Neglecting higher-order terms provides a linearized system of the form

(3) $$\left(\begin{array}{l} P_{1}\\ P_{2} \end{array}\right)=\left(\begin{array}{cc} a_{11} & a_{12}\\ a_{21} & a_{22} \end{array}\right)\left(\begin{array}{l} T\\ \sigma \end{array}\right).$$

where $a_{ij}$ represents the sensitivity coefficients forming the matrix A. If A is non-degenerate (detA≠0), the system admits a unique solution for *T* and *σ*.

However, if the sensor response is strongly nonlinear, Approximation (2) is valid only within a small neighborhood of $\left(T_{0},\sigma_{0}\right)$, limiting its practical use. Hence, one seeks a configuration in which the working range remains linear and the determinant of A remains sufficiently large to ensure stable inversion.

An analysis of the factors affecting the accuracy of reconstruction was carried out in [21]. In practice, two principal sources of error exist: (1) random errors in the measurement of $P_{1}$ and $P_{2}$; and (2) model errors related to inaccurate calibration of the coefficients $a_{ij}$ and to neglected nonlinear terms in the linearization from Equation (1) to Equation (3).

To study these effects, we consider the ideal case of Equation (3), which can be rewritten as

(4) $$\left\{\begin{array}{c} T=\frac{a_{22}P_{1}-a_{12}P_{2}}{\det A}\\ \sigma =\frac{a_{11}P_{2}-a_{21}P_{1}}{\det A} \end{array}\right\},$$

If the measured parameters contain small errors ∆*P*_1_ and ∆*P*_2_, these propagate into the estimated physical quantities as

(5) $$\left\{\begin{array}{c} \Delta T=\frac{a_{22}\Delta P_{1}-a_{12}\Delta P_{2}}{\det A}\\ \Delta \sigma =\frac{a_{11}\Delta P_{2}-a_{21}\Delta P_{1}}{\det A} \end{array}\right\}.$$

For an initially diagonal matrix (*a*_12_ = *a*_21_ = 0), Equation (5) simplifies to a trivial form: $\Delta T=\frac{\Delta P_{1}}{a_{11}},$$\Delta \sigma =\frac{\Delta P_{2}}{a_{22}}$, i.e., the greater the sensitivity coefficients $a_{11}$ and $a_{22}$, the smaller the relative errors in *T* and *σ*. However, for a non-diagonal matrix, cross-terms $a_{12}$ and $a_{21}$ introduce coupling between measurement channels and additional errors. As seen from Equation (5), the effective analogues of sensitivity are $\frac{\det A}{a_{22}}$ and $\frac{\det A}{a_{11}}.$ These “metrics” should be maximized when designing a two-parameter sensor to improve its stability and accuracy. Nevertheless, this analogy with the diagonal matrix is incomplete because cross-terms also produce correlated (cross-)errors in *T* and *σ.*

A numerical experiment was performed to analyze how the matrix coefficients ($a_{ij}$) influence the total measurement error. The procedure was as follows:

1. A sensitivity matrix *A* was generated with random elements.
2. The true values of *T* and *σ* were randomly assigned within a given range.
3. The exact values of *P*_1_ and *P*_2_ were computed using Equation (3).
4. Random measurement errors ∆*P*_1_ and ∆*P*_2_ were introduced within the specified range.
5. The exact (error-free) values of *T* and *σ* were calculated for ∆*P*_1_ = ∆*P*_2_ = 0.
6. The estimated *T* and *σ* were recalculated with the nonzero errors.
7. Individual measurement errors ∆*P*_1_ and ∆*P*_2_ were obtained.
8. Steps 2–7 were repeated 100 times, and the average error was determined.
9. The entire process was repeated from step 1 for newly generated matrices.

**Figure 1:**
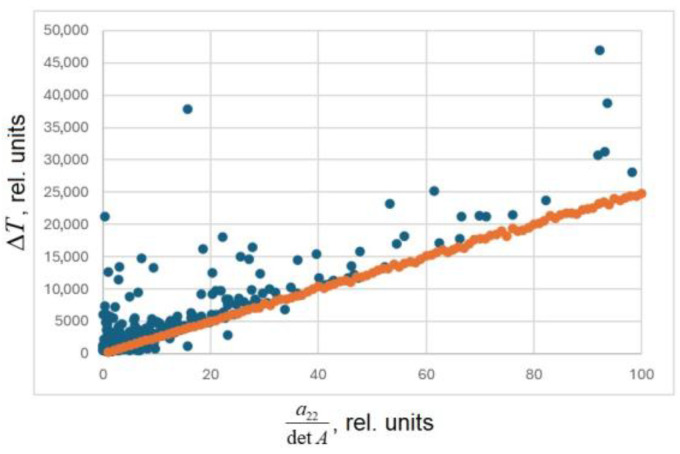
Dependence of the average temperature-determination error (in relative units) on the matrix parameter $\frac{a_{22}}{\det A}$ (in relative units). Orange markers denote diagonal matrices, illustrating independent parameter response, while blue markers correspond to non-diagonal matrices, where cross-correlation between parameters increases the estimation error.

The results are presented in Figure 1. From the figure, it follows that in the most favorable case of a diagonal matrix the errors directly correspond to the intrinsic sensitivities, whereas matrix non-diagonality introduces additional coupling and increases the total measurement uncertainty.

If the measured parameters of the sensor depend not only on *T* and *σ* but also on other physical influences, then two measured quantities are insufficient to separate all effects. In general, the number of measured parameters must be at least equal to the number of unknown physical quantities. In this case, a system of equations similar to Equation (1), but of higher dimension, is compiled, and the same procedure as described above is applied.

In addition to the direct analytical inversion of systems such as Equation (3), artificial-intelligence methods—especially machine-learning algorithms—have recently become increasingly popular for solving the cross-sensitivity problem. During the training stage, a dataset of correspondences between measured parameters (inputs) and known physical quantities (outputs) is provided to the algorithm. The system then learns the underlying relationships so that, during measurement, it can infer the most probable physical quantities directly from the sensor outputs.

A key advantage of this approach is the absence of an explicit calibration step, i.e., there is no need to experimentally determine the elements of matrix A, which can be technically difficult. Moreover, machine leaning models can capture nonlinear interactions and complex parameter coupling, which are often beyond the reach of simple linear models.

This section has presented the general theoretical foundations for separating the effects of multiple factors acting simultaneously on a sensing system. In practice, these principles—and the described process of separating influences—can be implemented in a variety of architectures. The most common fiber-optic sensor systems that rely on multiple physical effects include coherent optical frequency-domain reflectometers (C-OFDRs), coherent phase-sensitive optical time-domain reflectometers (Φ-OTDRs), and Brillouin optical time-domain reflectometers (BOTDRs).

Among these, Rayleigh scattering is the most intense in optical fibers, and the Φ-OTDR technique exploits this. Because Φ-OTDR systems are generally less complex and more cost-effective than BOTDR or C-OFDR implementations, this method is considered first in the following section.

## 3. Multi-Parameter Measurements with Φ-OTDR

### 3.1. The Principle of Φ-OTDR

Shortly after the development of low-loss optical fibers [22], optical time-domain reflectometry (OTDR) was proposed as a technique for testing long fibers with access to only one end [23]. Its operation relies on Rayleigh backscattering, which occurs naturally in optical fibers. A small fraction of the guided light is scattered in the backward direction and can be detected from the same end at which it was injected. Rayleigh scattering arises due to microscopic inhomogeneities in the fiber core—regions of fluctuating density *ρ* and refractive index *n* whose characteristic dimensions are much smaller than the optical wavelength *λ*. This process is elastic, meaning that it does not change the wavelength of light. Such inhomogeneities are unavoidable in fiber manufacturing: during the drawing of a silica-glass preform, local random variations in structure and composition become “frozen” into the fiber, producing refractive-index fluctuations that act as Rayleigh scattering centers. Quartz glass, used as the base material for most fiber preforms, is amorphous and behaves like a highly viscous liquid even in the solid state. Upon heating, molecular disorder increases, leading to local density fluctuations that alter both refractive index and dielectric constant. These thermodynamic fluctuations can be described, assuming entropy *s* and pressure *p* as independent variables, by the relation

(6) $$\Delta \rho ={\left(\frac{\partial \rho }{\partial p}\right)}_{s}\Delta p+{\left(\frac{\partial \rho }{\partial s}\right)}_{p}\Delta s$$

where *ρ* denotes the instantaneous deviation of density.

The second term represents the isobaric density fluctuations associated with entropy variations. Fluctuations in the dielectric constant *ε* are directly linked to density fluctuations [24]:

(7) $$\Delta \varepsilon ={\left(\frac{\partial \varepsilon }{\partial \rho }\right)}_{T}{\left(\frac{\partial \rho }{\partial s}\right)}_{p}\Delta s$$

Since the intensity of Rayleigh scattering is proportional to the number of scattering centers, it is also proportional to the variance of *ε*. The dielectric-constant fluctuations are, in turn, connected to entropy variations that follow the heat-conduction equation

(8) $$\rho c_{p}\frac{\partial \Delta s}{\partial t}-\kappa \nabla^{2}\Delta s=0,$$

where *k* is the thermal conductivity and *c_p_* is the specific heat capacity at constant pressure. In the temperature range relevant to fiber drawing, entropy *s* is approximately proportional to temperature *T*. After cooling, the structural fluctuations *n*(*z*) become “frozen” into the glass matrix, giving rise to a static distribution of Rayleigh scattering centers that determine the random backscatter pattern along the fiber.

In addition to Rayleigh scattering, light propagating in an optical fiber can undergo Brillouin and Raman scattering. Unlike Rayleigh scattering, these processes are inelastic, resulting in a wavelength shift in the scattered light that depends on the medium’s mechanical and thermal properties. One of the most important parameters governing light-matter interaction in such media is the polarization vector $\vec{P}$, which describes the response of the dielectric to the electric field $\vec{E}$:

(9) $$\vec{P}=\varepsilon_{0}\left(\chi^{\left(1\right)}\cdot \vec{E}+\chi^{\left(2\right)}\cdot \vec{E}\vec{E}+\chi^{\left(3\right)}\cdot \vec{E}\vec{E}\vec{E}+\ldots \right),$$

where $\varepsilon_{0}$ is the vacuum permittivity, and $\chi^{\left(j\right)}$ denotes the *j*-th-order susceptibility tensor of rank j+1. Entropy fluctuations do not propagate; therefore, from Equations (8) and (9), it follows that the linear susceptibility component $\chi^{\left(1\right)}\sim \Delta s$ produces a scattering term at the same frequency as the incident light corresponding to Rayleigh scattering. The intensity of Rayleigh scattering at a given point along the fiber thus depends on the local temperature, density, and concentration of scattering centers, all of which can be modulated by environmental disturbances. It also reflects the local optical loss of the medium. Consequently, the backscattered intensity varies along the fiber length. Because light scattered from different positions returns to the input end with different delays, the detected Rayleigh signal, known as the OTDR trace, represents optical power as a function of time. Knowing the group refractive index $n_{g}$ of the fiber core, the time coordinate t can be converted to spatial distance z [25]:

(10) $$z=\frac{c}{2n_{g}}t$$

where c is the speed of light in vacuum.

This conversion enables the localization of any external event or perturbation affecting the scattering intensity along the fiber. To avoid interference between signals scattered from different locations, light is launched into the fiber as pulses rather than continuously. The spatial resolution Δz, i.e., the minimum distinguishable fiber segment, depends on the optical pulse duration τ [25]:

(11) $$\Delta z=\frac{c}{2n_{g}}\tau$$

Thus, shorter optical pulses yield finer spatial resolution but at the expense of reduced signal power and sensitivity. This fundamental trade-off defines the operating characteristics of Φ-OTDR systems and forms the basis for their application in distributed sensing.

**Figure 2:**
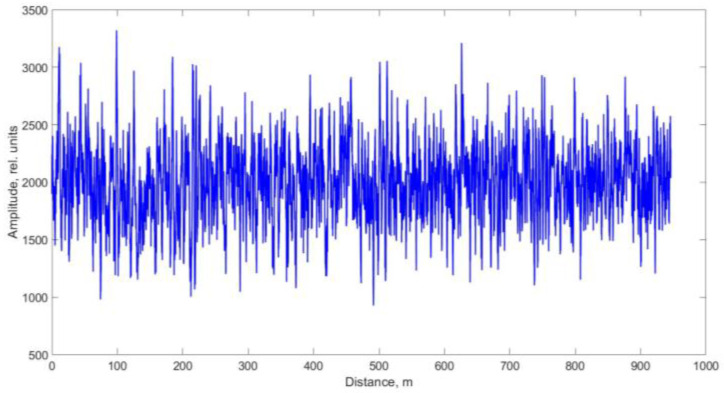
Example of a trace obtained using coherent optical time-domain reflectometry (COTDR).

In conventional OTDR, a large number of backscattered traces must be recorded and averaged to distinguish real events from background noise. This averaging, however, suppresses rapidly varying phenomena such as acoustic vibrations or shock waves. A qualitative breakthrough occurred in 1982 with the advent of Coherent OTDR (COTDR) [26]. In COTDR, a fraction of the continuous-wave (CW) laser radiation is diverted before pulse formation and then interfered with the Rayleigh-backscattered light returning from the fiber. The resulting signal resembles a standard OTDR trace but consists of numerous fine interference peaks (Figure 2).

The positions and amplitudes of these peaks depend on the relative distribution of Rayleigh scattering centers along the fiber. Their relative locations determine the phase of the scattered light during interference and, consequently, the instantaneous signal intensity. Because this interference pattern carries phase information, individual events can be detected against the noise background without averaging, which enabled the observation of fast dynamic processes and led to the creation of fiber-optic distributed acoustic sensors (DAS) based on Rayleigh scattering. Sound propagation in the medium can be represented as a periodic pressure variation Δ*P*. The application of acoustic pressure to the fiber produces periodic compressions and rarefactions, resulting in small displacements of scattering centers and, hence, phase modulation of the backscattered field. The optical phase sensitivity $s_{\phi }$ to pressure is given by

(12) $$s_{\phi }=\frac{\Delta \phi }{\phi \Delta P}$$

where ϕ is the optical phase and Δϕ its variation under external impact. Taking into account

(13) $$\phi =\frac{2\pi n_{g}}{\lambda }L$$

where L is the length of the fiber section exposed to acoustic pressure, the total phase change is expressed as

(14) $$\Delta \phi =\sigma L\left\{\beta -\frac{1}{2}\beta n^{2}\left[(1-\mu )p_{12}-\mu p_{11}\right]\right\},$$

where *β* is the propagation constant, *n* is the refractive index, *μ* is Poisson’s ratio, and *p*_11_ and *p*_12_ are the elasto-optic coefficients [27].

**Figure 3:**
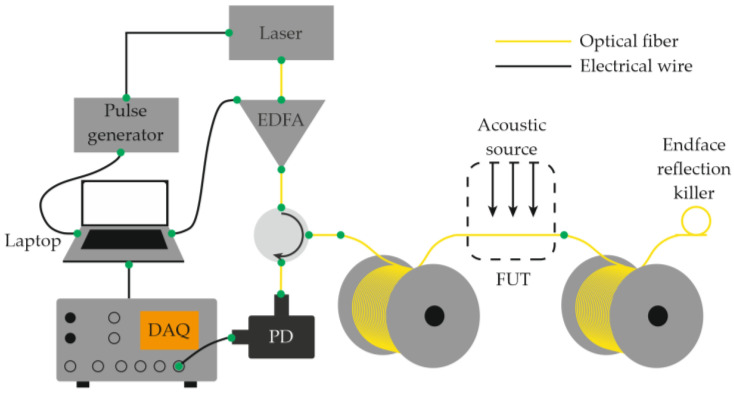
Basic Φ-OTDR setup schematic. Typical raw output data is presented in Figure 2. EDFA—erbium-doped fiber amplifier; PD—photodetector; FUT—fiber under test; DAQ—data acquisition card.

Equations (12)–(14) show that the ability to record acoustic effects through reflectometry depends on the magnitude of the sound pressure, the mechanical compliance of the fiber, and the length of the perturbed region. The use of a narrow-linewidth laser source with a long coherence length made it possible to eliminate the need for an external reference beam in the interferometric scheme. In this case, interference occurs directly inside the fiber between Rayleigh-scattered components within the optical pulse duration [28]. This simplification reduced the complexity of COTDR systems while preserving their fundamental operating principle and signal form. A basic Φ-OTDR setup schematic for distributed acoustic monitoring thus includes a laser source (continuous with an external modulator or pulsed through current control), an optical amplifier (if needed), a fiber-optic circulator or coupler, a sensing fiber, a photodetector, and a data-acquisition and processing unit (Figure 3) [29].

The quantitative development of these sensors became possible with the introduction of signal demodulation and phase-extraction techniques [30,31]. The resulting technology—phase-sensitive OTDR (Φ-OTDR)—has become the most widely used method for distributed acoustic sensing. Φ-OTDR-based sensors are now applied in a wide range of fields, including mineral exploration [32,33], oil and gas production, refining [34], and pipeline transportation [35,36,37], as well as the monitoring of civil infrastructure [38,39], transport systems [40], and perimeter security [28]. Emerging applications include agriculture [41], where distributed acoustic measurements are used to detect biological and environmental activity.

Despite their versatility, the broader deployment of Φ-OTDR systems remains limited, partly due to their relatively high cost. For some applications, however, this cost barrier can be mitigated by lowering the expense of the narrow-linewidth laser sources required for phase-sensitive detection [42,43]. Specifically, lasers based on self-injection locking have recently demonstrated excellent performance in Φ-OTDR configurations, providing narrow spectral linewidths, low frequency noise, and enhanced long-term stability [44,45,46,47] at only a fraction of the cost of conventional tunable or ultra-stable laser sources. These advances make self-injection-locked lasers a promising and practical solution for developing affordable, high-sensitivity distributed acoustic sensing systems.

In general, the Rayleigh scattering in each fiber segment is influenced by the local strain σ, which may originate from acoustic, mechanical, thermal, or even chemical perturbations. In certain situations, different types of effects can be discriminated by their frequency or amplitude signatures. Nevertheless, the simultaneous measurement and discrimination of multiple physical quantities using Φ-OTDR remains an active and rapidly evolving research area, which is continuing to attract considerable scientific and technological attention.

### 3.2. Multiparameter OTDR Sensing

In last decade, numerous approaches have been proposed to expand Φ-OTDR sensing beyond vibration detection and enable the simultaneous measurement of multiple physical quantities such as temperature, strain, and acoustic pressure.

**Figure 4:**
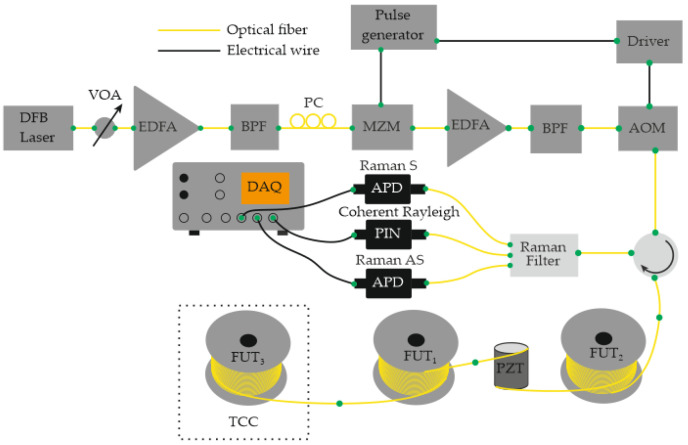
Setup diagram for simultaneous recording of thermal and acoustic impacts. DFB—distributed feedback laser; BPF—band-pass filter; MZM—Mach-Zehnder modulator; AOM—acousto-optic modulator; PZT—piezoelectric tube; TCC—thermally controlled chamber; APD—avalanche InGaAs photodetector; PIN—p-i-n photodetector; VOA—variable optical attenuator.

In 2016, Muanenda et al. [48] developed a hybrid distributed fiber-optic sensor capable of detecting acoustic and thermal perturbations simultaneously. A DFB laser with a linewidth of approximately 4 MHz produced a stable intra-pulse interference pattern and mutual incoherence between successive pulses. This allowed for efficient noise suppression while supporting cyclic pulse coding (Simplex) for multiparameter operation. To separate temperature and vibration signals, a Raman optical filter routed the Rayleigh, Raman Stokes, and Raman anti-Stokes components to three independent photodetectors (Figure 4). The system simultaneously achieved acoustic detection of up to 500 Hz and a temperature resolution of 0.5 °C at 0, 25, and 50 °C over 5 km of fiber with a 5 m spatial resolution.

**Figure 5:**
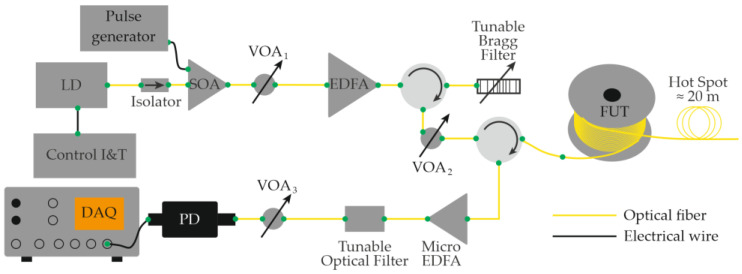
Schematic of a fiber-optic distributed acoustic sensor based on Φ-OTDR, enabling registration of temperature gradients through post-processing. LD—laser diode; SOA—semiconductor optical amplifier.

That same year, García-Ruiz et al. [49] demonstrated that a conventional single-frequency Φ-OTDR can be used to monitor distributed temperature gradients by analyzing each backscatter trace as a one-dimensional speckle structure. Unlike hybrid methods, this technique requires no laser frequency scanning, no heterodyning, and no additional hardware, relying solely on the post-processing of recorded traces. The experimental setup (Figure 5) was nearly identical to that used for distributed acoustic sensing (Figure 3). Using the “speckle-analysis” algorithm, the team measured a cooling gradient from 52.8 to 50.3 °C over 10 min with a polling rate of 1 trace per second. A stable SNR was maintained for gradients below 1 °C min^−1^. The spatial resolution reached 2 m, and the sensing range extended to 33 km of standard single-mode fiber.

**Figure 6:**
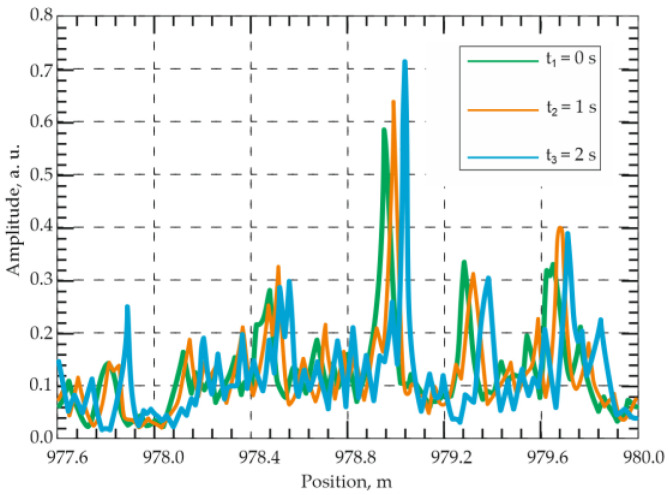
Spatial shift in Φ-OTDR traces relative to each other over time during heating.

Using the same hardware configuration (Figure 5), García-Ruiz et al. [50] later implemented simultaneous temperature and dynamic strain measurements by feeding a ramp signal to the laser to induce a linear frequency chirp. The chirped pulses eliminated the need for signal averaging, thereby enhancing temperature-measurement precision. Temperature variations were detected as spatial shifts between consecutive traces acquired at times t and t + 1 (Figure 6); no shift occurred under constant T. Simultaneously, dynamic strain was evaluated by comparing subsequent successive traces. The method is compatible with classical Φ-OTDR systems without major modification. In a 1 km experiment, 10 m spatial resolution was achieved. However, the authors claimed it was scalability with a spatial resolution of several meters and fiber length of several tens of kilometers. The reported resolutions were 1 mK for temperature and 4 nε for strain. The dynamic strain amplitude ranged from −300 to 300 nε at 1–850 Hz, and temperature tests involved heating a 20 m fiber section from 23 to 27.5 °C and cooling it over 270 min.

**Figure 7:**
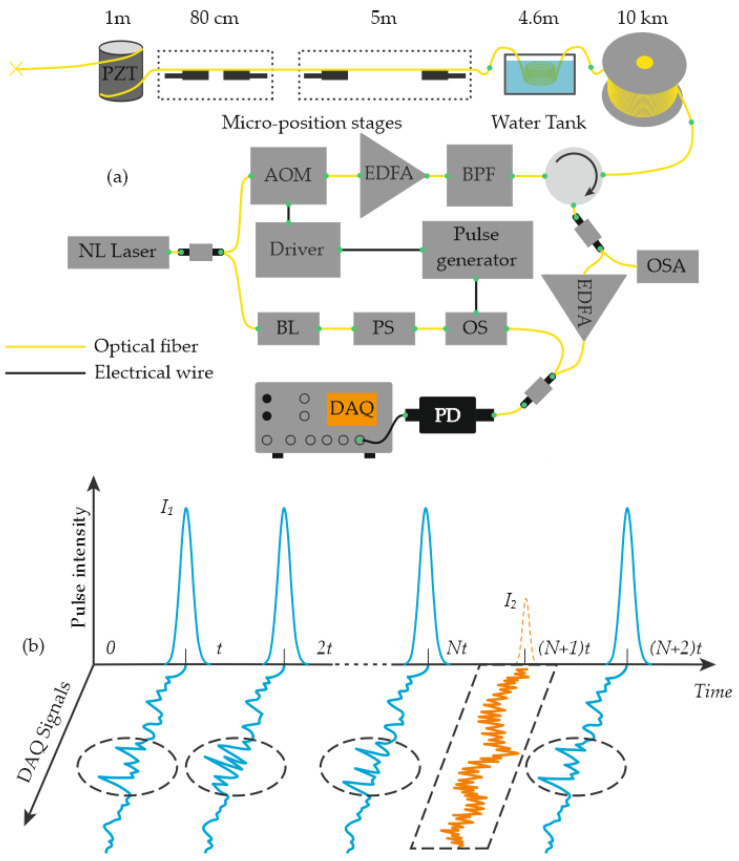
(**a**) Unit combining Φ-OTDR and BOTDR techniques for the simultaneous recording of temperature, vibration, and deformation effects. NL—narrow-linewidth laser; OSA—optical spectrum analyzer; OS—optical switch; PS—polarization scrambler; BL—Brillouin laser. (**b**) Probe pulses used in the device and a representative sample of the recorded output data.

Zhang and co-authors proposed a hybrid scheme that combines Φ-OTDR and BOTDR to simultaneously measure temperature, vibration, and strain [51]. In their setup, a single modulator, one photoreceiver, and one DAQ channel are used with standard SMF (Figure 7a). The probe sequence is time-multiplexed by tailoring pulse width and peak power to the target phenomenon: broad, high-intensity pulses capture rapidly varying vibrations (Figure 7b), whereas lower-intensity pulses with a longer repetition period are allocated to the more slowly varying temperature *T* and strain σ channels. Signal processing employs an FFT with a Gaussian window. This design enables the simultaneous recording of vibrations up to 4.8 kHz with ~3 m spatial resolution, and distributed *T* and *σ* profiles with ~80 cm spatial resolution over 10 km of fiber. In experiments, T varied from 24.8 to 72.5 °C, and applied strain reached 2000 µε.

**Figure 8:**
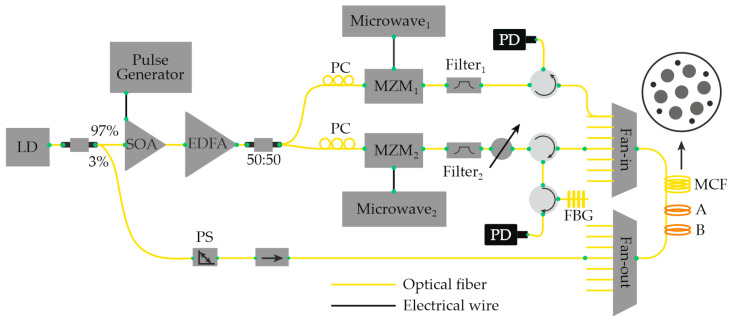
Diagram of a setup combining BOTDR and Φ-OTDR using multicore fiber (MCF). PC—polarization controller; PS—polarization switch.

In 2017, Dang et al. [52] demonstrated a hybrid distributed-sensing architecture that unites the advantages of Φ-OTDR and BOTDR within a single system. The approach employed a multi-core fiber (MCF) in which the two measurement channels were spatially separated: Φ-OTDR pulses were launched into one of the outer cores, while the Brillouin channel operated through the central core (Figure 8). This configuration simultaneously provided the wide dynamic range typical of BOTDR and the high spatial and temperature-strain resolution characteristic of Φ-OTDR, without requiring separate laser sources or optical amplifiers. A single electro-optic modulator was sufficient to generate the probe pulses for both channels. Under these conditions, the achievable spatial resolution for each method was approximately 2.5 m over a sensing length of 1.565 km. The hybrid system demonstrated a temperature resolution of about 0.001 °C, with temperature variations measured between 16 °C and 81 °C. The authors emphasized that both Φ-OTDR and BOTDR responses are sensitive to temperature and mechanical strain (acoustic perturbations). Although this particular work did not attempt to separate these influences, several referenced studies were cited as potential strategies for future discrimination of the two effects.

**Figure 9:**
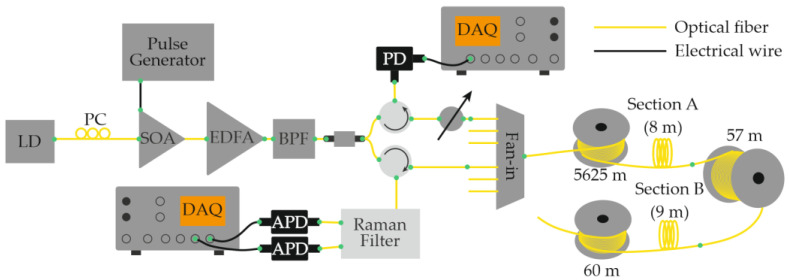
Experimental setup combining Raman and Rayleigh reflectometry for the simultaneous detection of thermal and acoustic effects.

Building on the concept of hybrid scattering mechanisms, in 2018, Zhao et al. [53] proposed another configuration that combines Φ-OTDR and Raman OTDR (ROTDR) in a single distributed sensor. A key challenge in such systems arises from the fact that the power of spontaneous Raman scattering is typically about 30 dB lower than that of Rayleigh scattering. To ensure sufficient Raman signal strength, high pump power is required—however, this can introduce nonlinear effects and distort the Φ-OTDR trace. To overcome this incompatibility, the authors implemented the system using a multi-core fiber (MCF), which provides spatial separation of the Rayleigh and Raman channels. The laser source, optical amplifier, and pulse-modulation stages were shared between both subsystems. After modulation, the optical pulse was split into two parts: one was attenuated and launched into an outer core of the MCF to generate the Rayleigh backscattering signal, while the other was directed into the central core for Raman sensing (Figure 9). This design ensured the independent detection of Rayleigh- and Raman-scattered light, preventing nonlinear interference and allowing for the simultaneous measurement of acoustic and temperature variations with spatial discrimination of the sensing channels. As a result, the Rayleigh and Raman scattering signals in the hybrid system were successfully discriminated and detected independently. Experimental results further showed that the outer cores of the multi-core fiber were more sensitive to bending than the central core. Consequently, employing the outer cores for recording acoustic and deformation signals via Φ-OTDR, while reserving the central core for Raman-based temperature sensing, proved to be an effective strategy. Measurements were performed at temperatures of 50 °C, 60 °C, and 75 °C, demonstrating the simultaneous detection of acoustic and thermal effects with a spatial resolution of 3 m over a fiber length of 5.76 km.

**Figure 10:**
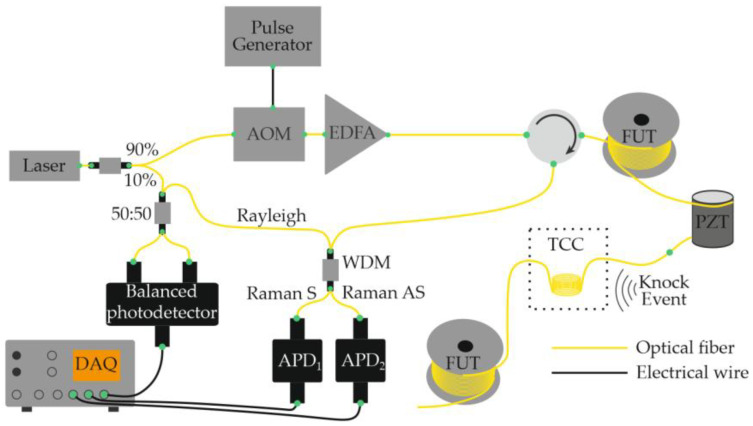
Diagram of a hybrid setup combining Raman and Rayleigh reflectometry for the simultaneous reconstruction of acoustic and temperature fields.

In the same year, another research group presented a hybrid fiber-optic distributed sensor capable of the simultaneous reconstruction of acoustic and temperature fields [54]. A key feature of this study is that thermal and acoustic effects were applied simultaneously to the same section of the optical fiber (Figure 10). As in the configuration proposed by Muanenda et al. [48], the Rayleigh and Raman scattering components were separated prior to detection and directed to individual photodetectors. The authors reported that a probing pulse with a peak power of 22 dBm, pulse width of 100 ns, and repetition rate of 6 kHz provided a satisfactory compromise between spatial resolution and signal-to-noise ratio for both temperature and acoustic measurements. The experiments were conducted using a standard single-mode fiber, and no significant nonlinear effects, such as stimulated Raman scattering or modulation instability, were observed. The system achieved a temperature-measurement accuracy of 0.95 °C for heating between 35 °C and 55 °C in 5 °C increments, while acoustic signals were detected across the 100–1000 Hz frequency range. The spatial resolution was 10 m for both temperature and acoustic sensing over a fiber length of 12 km.

In 2019, Hicke et al. [55] reported a set of results demonstrating several improvements relevant to the functionality and performance of distributed fiber-optic vibration sensors. These advances were achieved using a standard single-mode fiber (SMF) modified to contain uniformly distributed artificial scattering points inscribed by a femtosecond laser. This modification is particularly advantageous for traditional coherent OTDR (COTDR) systems. The inscribed scatterers form quasi-distributed interferometric sensing zones, defined by the fiber segments between successive scattering points. Within these zones, the local sensitivity to external perturbations is enhanced, and the otherwise nonlinear transfer function of the system becomes effectively linearized, resembling that of a conventional dual-beam interferometer. This linearization improves the accuracy of phase response to monotonic temperature variations. Additionally, the modification reduces sensitivity fading and enables low-frequency demodulation.

An important advantage of this approach is that it allows for the direct quantification of both local temperature gradients and acoustic effects from the intensity of the Rayleigh backscatter trace. The reflectivity of the artificial scattering points, and thus the overall optical attenuation, can be controlled by adjusting the laser-inscription parameters. This provides a simple, reliable, and cost-effective method to enhance sensor performance without requiring complex interrogation systems or the fabrication of ultra-weak fiber Bragg grating (UWFBG) arrays. In the experiments, a commercial interrogation unit (Helios HSI, Fotech Solutions Ltd., Hampshire, UK), originally designed for distributed acoustic sensing, was used instead of a laboratory prototype. The modified fiber and processing method enabled the simultaneous detection of vibration and temperature gradients with a spatial resolution of 10 m over a 55 km sensing range. During temperature measurements, the fiber was heated from 24.7 °C to 31.2 °C, yielding a temperature accuracy between 0.038 and 0.4 °C. The lowest detectable vibration frequency was 5 Hz. The distance between the artificial scattering points was 6.48 m, slightly shorter than the pulse length of the probe light, thereby avoiding a transition from distributed to quasi-distributed operation and maintaining continuous sensing capability.

**Figure 11:**
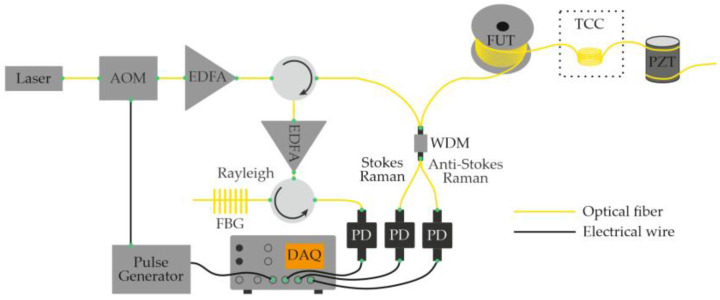
Setup for the simultaneous distributed monitoring of temperature and vibration using a multimode optical fiber operated in a quasi-single-mode regime.

In 2020, Mao et al. [56] demonstrated the simultaneous monitoring of temperature and vibration using a multimode optical fiber (MMF) operated in a quasi-single-mode regime. By exciting only the fundamental mode of the MMF, the authors effectively combined distributed temperature and distributed vibration sensing within a single platform: two measurement modalities that are typically difficult to merge due to their differing operational requirements. The study showed that operating the multimode fiber in a quasi-single-mode regime provides significant advantages compared to both the true multimode operation of the same fiber and the use of a standard single-mode fiber (SMF). As in several previous works, the Rayleigh and Raman (Stokes and anti-Stokes) scattering components were spectrally separated before detection, and each was directed to a dedicated photodetector (Figure 11). The system successfully recorded temperatures of 7.2 °C and 48.3 °C with an accuracy of 1 °C, while simultaneously detecting acoustic vibrations at 5 kHz with an average signal-to-noise ratio (SNR) of 13 dB. The spatial resolution was 10 m, and the fiber length used in the experiments was 4 km.

As a related advancement, the same research team later introduced a configuration based on low-mode fiber [57], designed for the simultaneous measurement of temperature and acoustic effects. The system architecture was nearly identical to that shown in Figure 11, differing only in fiber type. Experiments carried out along 3.1 km of low-mode fiber placed in an indoor environment (at 22 °C) demonstrated successful detection of a vibration event at 200 Hz. The spatial resolution remained 10 m for both temperature and vibration monitoring.

In 2024, Wang et al. [58] proposed and experimentally demonstrated a distributed fiber-optic sensor based on Φ-OTDR with frequency scanning, capable of the simultaneous measurement of salinity and temperature. The authors employed an anisotropic, polarization-maintaining optical fiber coated with polyimide, a hygroscopic material that converts variations in environmental salinity into the mechanical deformation of the fiber. Due to the fiber’s birefringence, the Rayleigh backscattering frequency shift, analogous to that observed in OFDR systems, exhibits different sensitivities to temperature and salinity along the slow and fast polarization axes. This property enables the two parameters to be discriminated using a single sensing fiber. In the experiments, pulse sequences with orthogonal polarization states were alternately launched into the fiber to probe both axes. This design overcame the limitations of the team’s earlier single-mode, polyimide-coated salinity sensor [59], which could not fully decouple temperature and salinity influences in the output signal. The improved configuration achieved measurement accuracies of 0.0344 K for temperature and 0.0469 mol·L^−1^ for salinity. The temperature sensitivities were 1407.8 MHz·K^−1^ for the slow axis and 1348.9 MHz·K^−1^ for the fast axis, while the corresponding salinity sensitivities were 1028 MHz/(mol·L^−1^) and 1008.6 MHz/(mol·L^−1^), respectively.

These results demonstrate that polarization-resolved Φ-OTDR with hygroscopic fiber coatings provides a powerful route to dual-parameter environmental sensing in aqueous or humid environments.

In the same year, Huang et al. [60] proposed and experimentally demonstrated a compact hybrid fiber-optic distributed sensing system capable of simultaneous vibration and temperature measurements. The system design is notably simplified, utilizing only two signal channels, which enhances its practicality and integration potential. Based on a conventional OTDR architecture, the setup separates Rayleigh-scattered light and Raman anti-Stokes scattering to perform vibration and temperature sensing, respectively. For vibration detection, the authors developed a differential location search algorithm that analyzes the polarization state of the Rayleigh backscatter. This algorithm first compensates for fiber attenuation in the original trace to equalize the signal level along the sensing length, and then determines the position of acoustic events by evaluating trace-to-trace differences followed by threshold discrimination. For temperature measurements, a dedicated temperature calibration unit and dynamic correction algorithm were introduced to minimize errors caused by laser-pulse instability. The system achieved a temperature measurement range from −0.7 °C to 1.3 °C with an error of 0.85 °C. For both vibration and temperature measurements, the spatial resolution was 6 m, and the sensing fiber length reached 12 km.

**Figure 12:**
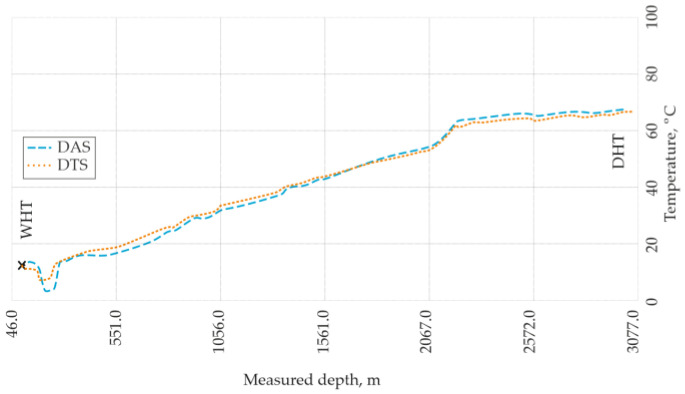
Comparison of distributed acoustic and distributed temperature sensor data obtained during well temperature measurements.

In the same year, Bradley et al. [61] proposed a practical approach for discriminating temperature and acoustic influences in distributed acoustic sensing (DAS) during well monitoring. The authors suggested selecting quiet operational periods to minimize acoustic activity and operating the DAS system in a low-frequency mode to further suppress interference from sound and mechanical deformations. In this configuration, the polling rate was set to approximately 1.25 Hz, corresponding to a maximum detectable acoustic frequency of about 0.6 Hz. Such a regime effectively reduces cross-sensitivity to vibration and other deformation-induced effects when performing temperature measurements in well environments. A comparative field experiment demonstrated strong agreement between data obtained from a dedicated distributed temperature sensing (DTS) system and temperature readings derived from the low-frequency DAS configuration under real well conditions (Figure 12). Despite minor residual deformation-related noise, the results clearly validated the efficiency and accuracy of the proposed method for temperature discrimination in distributed acoustic sensors.

In conclusion, it is useful to briefly summarize the most significant advances in the discrimination and simultaneous measurement of temperature, acoustic, and strain effects in distributed acoustic sensors (DAS) and Φ-OTDR systems. The key achievements from recent studies are compiled in Table 1.

Based on these results, several general observations can be made. The primary challenge for most research groups remains the discrimination and concurrent measurement of acoustic and thermal influences in Φ-OTDR systems. Existing discrimination strategies can typically be categorized into four major groups:

(1) Spatial separation—achieved using specialized fibers such as multi-core, multimode, low-mode, or anisotropic fibers.
(2) Temporal separation—based on time-gated measurements, which inherently prevent truly simultaneous multi-parameter acquisition.
(3) Spectral or frequency (wavelength) separation—implemented through wavelength-division multiplexing or by exploiting different scattering mechanisms (Rayleigh, Brillouin, Raman).
(4) Software-based discrimination—involving advanced signal processing, statistical analysis, or machine-learning-assisted data interpretation.

Each approach offers specific advantages but also inherent trade-offs. For instance, Rayleigh-only sensing architectures generally provide high accuracy yet are limited to a narrow temperature range. Hybrid systems that incorporate Brillouin or Raman scattering extend the dynamic range of measurable quantities but at the cost of increased system complexity and hardware requirements.

Recent research has also broadened the range of measurable parameters beyond strain and temperature. A notable example is the development of salinity-sensitive fiber sensors that utilize hygroscopic coatings to convert environmental conditions into measurable strain-induced shifts in the Rayleigh spectrum. Such innovations illustrate the expanding scope of Φ-OTDR-based multiparameter sensing toward chemical and environmental monitoring. Overall, the reviewed studies confirm that Φ-OTDR remains a highly active and rapidly evolving research area, particularly regarding multi-quantity discrimination. Among the reported methods, the approach by García-Ruiz et al. [44] is especially notable for achieving dual-parameter (temperature and strain) measurement without complex hardware modifications or computationally intensive algorithms. Similarly, hybrid configurations combining Φ-OTDR and BOTDR have demonstrated the feasibility of three-parameter sensing (temperature, strain, and vibration) using coordinated interrogation schemes. Each of these architectures, though distinct, represents a rational and practical solution suited to specific monitoring applications.

A fundamental limitation of Φ-OTDR technology arises from the direct proportionality between spatial resolution and optical-pulse duration. Shortening the probe pulse improves spatial resolution but reduces the effective sensing length. Conversely, increasing the pulse intensity to maintain signal power eventually triggers inelastic scattering processes—namely, Brillouin and Raman scattering—which introduce unwanted nonlinearities and complicate data interpretation. This limitation can be overcome by employing continuously tunable light sources, which eliminate the dependence of spatial resolution on pulse duration. However, such sources significantly increase system complexity and cost. The corresponding technology, known as Optical Frequency-Domain Reflectometry (OFDR), is inherently sensitive to temperature variations as well as slow and fast mechanical deformations. The principles and capabilities of multiparameter sensing using OFDR are discussed in detail in the following section.

## 4. Simultaneous Measurement of Multiple Physical Quantities Using OFDR

### 4.1. The Principle of OFDR

Optical Frequency-Domain Reflectometry (OFDR) has gained wide application not only in fiber-optic metrology but also in distributed fiber-optic sensing [62,63]. Its main advantages are ultra-high spatial resolution and sensitivity to temperature *T* and strain *σ*. Before discussing advanced techniques for separating these parameters, it is essential to review the operating principle of OFDR and the fundamental method for extracting the relevant physical quantities from the measured data. The operating concept of OFDR originates from the frequency-modulated continuous-wave (FMCW) principle used in radar systems. In both cases, a signal of linearly swept optical frequency is mixed with a reference signal, producing a beat frequency that carries information about the optical path difference between two arms of an interferometer. In fiber optics, this heterodyne detection method allows high-resolution reflectometry without the need for high-power pulsed excitation, as used in classical OTDR. Moreover, because OFDR records both amplitude and phase of the backscattered field, it enables retrieval of local strain or temperature perturbations by comparing the instantaneous trace to a reference measurement.

**Figure 13:**
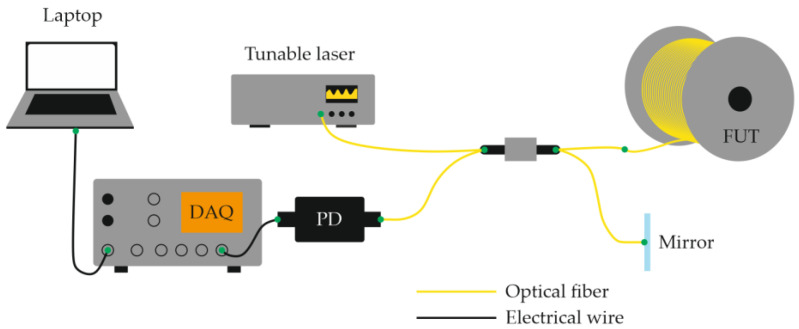
Basic OFDR schematic. Typical output data is shown in Figure 14.

A schematic of a basic OFDR system is shown in Figure 13. Radiation from a tunable laser source (TLS) is divided by an optical coupler into reference and measurement arms. The reference arm provides a stable phase reference, while the other launches light into the fiber under test (FUT). Due to microscopic fluctuations in the refractive index, the fiber produces Rayleigh backscattering continuously along its length. The backscattered field from each location interferes with the reference field at the detector, forming a composite interference fringe whose beat frequencies are proportional to the distances of the scattering sites.

The detected signal reflected from two arms can be represented as the superposition of two fields with slightly different optical frequencies. The interference term can be expressed as

(15) $$X=2\cdot A\cdot B\cdot \cos\left(\frac{\Delta \omega \cdot t}{2}\right)\cdot \cos\left(\omega \cdot t\right),$$

where *A*, *B* are the amplitudes of the interfering signals, Δω is the instantaneous frequency difference in the interfering signals, and ω is the optical carrier frequency.

Neglecting noise, the electric field of the linearly tuned laser is

(16) $$E\left(t\right)=E_{0}\exp\left[i\left(2\pi \upsilon_{0}+\pi \gamma t^{2}\right)\right],$$

where *E*_0_ is the amplitude of the laser field, *i* is the imaginary unit, $\upsilon_{0}$ is the initial frequency, and γ is the speed of the laser frequency tuning.

As the optical field propagates through the FUT, it experiences partial reflections from localized refractive-index perturbations (Rayleigh scattering centers). Each reflection is characterized by a local reflection coefficient $r_{i}$ and a corresponding time delay $\tau_{i}$ that defines the scattering position (in respect to the mirror position). The total backscattered field is then the coherent sum of all individual reflections:

(17) $$E_{F}\left(t\right)=\sum_{k}r_{k}E_{0}\exp\left(i\left[2\pi \upsilon_{0}\left(t-\tau_{k}\right)+\pi \gamma {\left(t-\tau_{k}\right)}^{2}\right]\right).$$

The recorded signal is the result of the interference of the laser reference signal and the signal backscattered from the fiber under study. At the detector, the interference between the reference field and the scattered field yields the measured signal intensity:

(18) It=ReEt×EF∗t=∑kriE02cos2πγτit+2πυ0τi−πγτk2,

where (*) denotes the complex conjugate and Re{ } extracts the real part. Each spectral component of the interference pattern corresponds to one scattering site along the fiber; thus, the beat frequency directly maps to the distance of that site.

**Figure 14:**
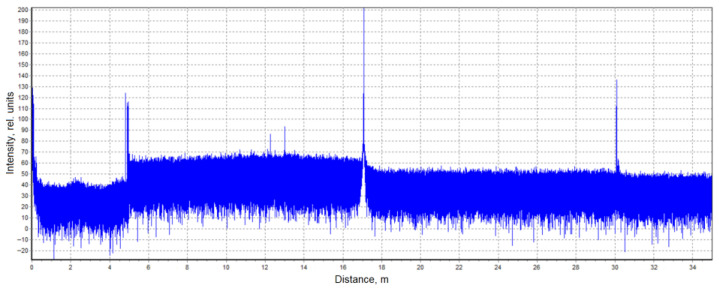
Example of a trace obtained using optical frequency-domain reflectometry (OFDR).

Applying a Fourier transform to *I*(*t*) converts the time-domain interference pattern into a spatial-domain reflectogram (or OFDR trace). For a linear frequency sweep $\nu \left(t\right)=\nu_{0}+\alpha t$ to the *k*-th scattering center is related to its beat frequency $f_{k}$ as

(19) $$l_{k}=\frac{c}{2n(f_{2}-f_{1})}k,$$

where *c* is the speed of light in vacuum and $n_{g}$ is the group refractive index of the fiber. The resulting dependence of backscattered intensity *I*(*z*) on distance *z* constitutes the OFDR trace (Figure 14).

The random “frozen-in” refractive-index variations in the optical fiber form a stable Rayleigh scattering pattern, which can be modeled as a weak random Bragg grating. In the absence of external perturbations, this pattern remains stationary. External effects such as temperature *T* or strain *σ* shift the scattering centers, modifying the local propagation constant and thus the optical path length. The resulting phase or spectral displacement of the interference pattern can be expressed as

(20) $$\frac{\Delta \lambda }{\lambda }=K_{\varepsilon }\sigma +(\alpha_{L}+\alpha_{n})\Delta T,$$

where $K_{\varepsilon }$ is the strain sensitivity coefficient, $\alpha_{L}$ is the linear thermal expansion coefficient, and $\alpha_{n}$ is the thermo-optic coefficient.

The corresponding optical phase shift due to mechanical elongation of the fiber can be approximated by

(21) $$\Delta \varphi =\frac{4\pi n\Delta L}{\lambda }.$$

where ΔL is the change in physical length. However, strain also modifies the effective refractive index via the photoelastic effect, as described previously by Equation (14).

The temperature-induced phase shift arises from two mechanisms: (1) change in refractive index (thermo-optic effect), and (2) change in physical length (thermal expansion). This can be expressed as

(22) $$\Delta \varphi_{T}=\Delta \varphi_{T}\left[dn\left(T\right)+dl\left(T\right)\right]$$

The dependence of the refractive index of fused silica on temperature is empirically provided by [64]

(23) $$dn\left(T\right)=\frac{1}{n}\left(\frac{\partial n}{\partial T}\right)=0.68\cdot 10^{-5}\;{{}^{\circ}C}^{-1}$$

(24) $$\Delta \varphi \left(n\right)=\frac{2\pi l\gamma (t-\tau )}{c}\frac{1}{n}\left(\frac{\partial n}{\partial T}\right),$$

where *l* is the length of the section of change in fiber temperature.

Taking these effects together, the total optical path change under temperature variation is

(25) $$\Delta \varphi \left(n\right)=0.68\cdot 10^{-5}\cdot \frac{2\pi l\gamma (t-\tau )}{c}\Delta T.$$

and the physical fiber elongation caused by thermal expansion is

(26) $$\Delta l\left(T\right)=\alpha_{T}\Delta T,$$

(27) $$\Delta \varphi \left(T\right)=\frac{n\gamma (t-\tau )}{c}l\alpha_{T}\Delta T.$$

where $\alpha_{T}$ is the thermal expansion coefficient of fused silica, typically below 10^−6^ °C^−1^ across a wide temperature range.

In addition to phase or spectral information, the amplitude envelope of the OFDR trace contains valuable data about linear optical losses in the fiber under test. The local backscattered power *P*(*z*) along the fiber decreases exponentially with propagation distance between two fiber points $z_{1}$ and $z_{2}$ according to $P(z_{2})=P(z_{1})\exp\left(-2\int_{z_{1}}^{z_{2}}\alpha \left(z\right)dz\right)$, where $\alpha \left(z\right)$ is the local attenuation coefficient of the FUT. Plotting lnP(z) versus distance yields a curve, which allows for the distribution of the linear loss coefficient along the FUT from the OFDR trace to be reconstructed.

This quantitative attenuation analysis offers additional capabilities for multiparameter sensing, enabling the simultaneous retrieval of distributed temperature, strain, and optical loss profiles along the fiber. It has proven particularly valuable for gamma-radiation dosimetry, where local variations in optical power caused by Radiation-Induced Absorption (RIA) in doped fibers are proportional to the accumulated dose, thus providing an additional diagnostic channel complementary to temperature and strain measurements [65,66,67,68].

### 4.2. Method of Correlation of Rayleigh Scattering Spectra

**Figure 15:**
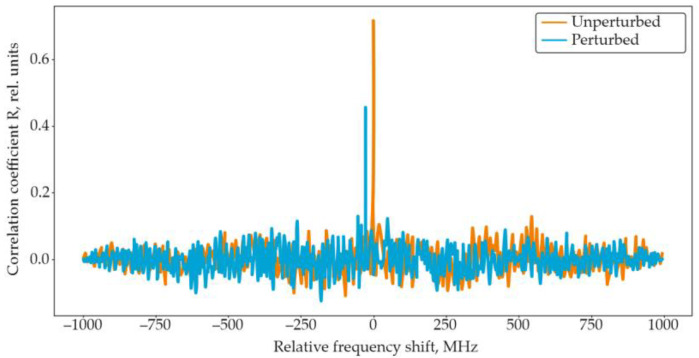
Correlation function of the Rayleigh backscattering signal without external impact (orange) and with external impact (blue).

A classical approach to demodulating the influence of strain σ in OFDR systems is based on measuring the spectral shift in the Rayleigh backscattering trace using a windowed correlation technique. The reference and perturbed OFDR traces—corresponding to the initial and measured states of the fiber—are divided into *N* equal segments, or windows, each containing n sampling points. An inverse Fourier transform is then applied to each window to convert the corresponding spectral segment into the time domain. The resulting signals from windows with identical indices are compared by calculating their cross-correlation function (CCF):

(28) $$CCF\left(n\right)=\sum_{k=-n}^{n-1}R_{i}\left[k\right]M_{i}\left[k+n\right],$$

where *R_i_* and *M_i_* are the signals in the i-th window for the reference and measured states, respectively; *k* is the relative shift between the two signals, and *n* is the window length. In OFDR systems, the Pearson correlation coefficient is typically used to normalize the cross-correlation function, which can be written as

(29) $$\rho_{R,M}=\frac{CCF\left(R_{i},M_{i}\right)}{\sigma_{R_{i}}\sigma_{M_{i}}},$$

where *R_i_* and *M_i_* are the mean values, and $\sigma_{R_{i}}$ and $\sigma_{M_{i}}$ are the variances in *R_i_* and *M_i_*, respectively. The resulting cross-correlation function exhibits a distinct peak at the point corresponding to the relative displacement between the two spectral fragments (Figure 15).

The measured spectral (or temporal) shift in this correlation peak is directly related to changes in strain or temperature, and can be expressed as

(30) $$\Delta \sigma =\frac{2\pi \gamma \Delta t}{0.78\omega_{0}}=\frac{2\pi \Delta \omega }{0.78\omega_{0}}$$

(31) $$\Delta T=\frac{2\pi \gamma \Delta t}{(\alpha_{L}+\alpha_{n})\omega_{0}}=\frac{2\pi \Delta \omega }{(\alpha_{L}+\alpha_{n})\omega_{0}}$$

This windowed cross-correlation technique remains the most widely used and robust method for the quantitative demodulation of Rayleigh scattering spectra in OFDR-based distributed sensors.

### 4.3. Discrimination of Various Fiber Impact Types Using OFDR

This section reviews the evolution of methods for separating the effects of different physical quantities—primarily temperature *T* and strain *σ*—in OFDR-based distributed sensors. The discussion begins with early works demonstrating separation through the use of different fiber types, cable configurations, and polarization modes of anisotropic fibers, and concludes with modern hybrid techniques that combine multiple physical principles and emerging machine-learning-based approaches capable of providing interpretable parameter discrimination. In OFDR sensing, the measurable quantity is typically a spectral (or correlation-function) shift, which can be influenced by both temperature and strain. Since both effects contribute to the same observable parameter, direct discrimination between them is not straightforward. To uniquely determine both *T* and *σ*, two independent parameters must be extracted from the measurement data.

#### 4.3.1. Discrimination of Two Physical Quantities Using OFDR

One of the earliest practical solutions to this problem was developed by researchers at Luna Innovations, culminating in a patent describing a method for distinguishing temperature and strain using an anisotropic optical fiber as the sensing element [69]. This pioneering work, later discussed in detail in a comprehensive review of recent OFDR advances [70], demonstrated that the polarization-dependent Rayleigh response in birefringent fibers could be exploited for dual-parameter discrimination. At the time of publication, the system achieved a temperature accuracy of ±3.5 °C and a strain accuracy of ±35 µε at the end of an 8.55 m sensing line. These were remarkable results for early OFDR technology.

Since then, substantial progress has been made: detector sensitivity has improved, laser frequency tuning has become more precise, and advanced signal-processing techniques, including machine-learning algorithms, have been introduced to enhance accuracy and robustness. In a subsequent study [71], researchers demonstrated that using two fibers with distinct structural properties—a standard single-mode fiber (SMF) and an SMF with a reduced cladding diameter—significantly improves measurement precision. Because these fibers exhibit different thermo-optic and strain-optic responses, their combination enables parameter decoupling through comparative analysis. The achieved accuracies were 0.3 K for temperature and 8 µε for strain over a 50 m sensing length, with a spatial resolution of 18 cm.

Another notable advancement was reported in [72], where the authors employed a bandwidth-division multiplexing scheme instead of a conventional 50/50 optical splitter. This configuration allowed for separation of the spectral responses associated with temperature and strain variations. The achieved measurement accuracies were 1.4 K for temperature and 20 µε for strain, with a spatial resolution of 10 cm.

Another promising and relatively recent approach to parameter discrimination involves the use of polarization-maintaining fibers (PMFs) instead of conventional single-mode fibers (SMFs) [73]. In this configuration, an additional parameter that characterizes the detected signal beyond the commonly analyzed Rayleigh backscattering (RBS) spectral shift is the RBS dispersion, which arises due to the propagation of light along the orthogonal fast and slow axes of the birefringent fiber. As demonstrated in the initial experiments, this dispersion parameter can be accurately retrieved by analyzing the autocorrelation function of the Rayleigh spectrum, specifically by locating the maximum correlation peaks corresponding to the interference between the optical waves propagating along the two birefringent axes. The combination of RBS bias (spectral shift) and RBS dispersion provides two independent observables that can be mapped to the physical quantities of temperature T and mechanical strain σ according to relations analogous to Equations (30) and (31). The experimental setup reported in [73] achieved exceptionally high accuracy, with a temperature deviation of ±0.1 K and a strain error of approximately 1 µε, over a 180 m sensing length and a spatial resolution of about 2.5 mm. Notably, this approach closely follows the principles described in the early Luna Innovations patent [69], yet significantly surpasses it in accuracy. Such outstanding results likely stem from the integration of established optical concepts with modern high-stability components and refined signal-processing algorithms.

A completely different methodology was proposed by Song et al. [74], targeting the discrimination of simultaneous vibration signals of different origins. Instead of separating the influences of temperature and strain, the authors designed an OFDR-based configuration capable of simultaneously detecting multiple overlapping vibration frequencies occurring at the same spatial location. The system employed a frequency-tunable laser with a constant central wavelength, enabling precise phase-resolved detection. Experimental verification demonstrated the method’s ability to identify and distinguish vibration signals at 50 kHz and 20 kHz, both reliably detected over a fiber length of 8 km. The achieved frequency resolution was approximately 1.26 kHz, and the spatial uncertainty of event localization was below 12 m. These results highlight the strong potential of phase-resolved OFDR correlation analysis for multi-frequency vibration discrimination, offering new opportunities for the distributed monitoring of complex dynamic environments.

#### 4.3.2. Discrimination of Three or More Physical Quantities Using OFDR

Building on these developments, the researchers in [75] expanded the concept of multiparameter OFDR sensing by introducing environmental humidity as an additional target parameter, alongside temperature and strain. They developed a specialized fiber sensor fabricated from high-strength birefringent fiber coated with a protective polyimide layer, designed to enhance both mechanical robustness and hygroscopic sensitivity. The sensor demonstrated the ability to measure ambient temperature with an accuracy of approximately ±0.5 °C and relative humidity with an error below 3%. The study revealed that changes in environmental humidity lead to mechanical deformation of the fiber cladding, which alters the local stress distribution and modifies the transmission characteristics of light through the fiber. These variations manifest as distinct spectral changes in the Rayleigh backscattering (RBS) signal. However, temperature fluctuations produce similar spectral deviations by affecting both the refractive index and physical length of the fiber. Consequently, accurate discrimination between humidity- and temperature-induced effects requires a comprehensive analysis of multiple spectral components of the measured data.

A major step forward was presented by Madry et al. in [76], who developed a hybrid OFDR-based sensor combined with a machine-learning algorithm to enable real-time and fully automated temperature and humidity demodulation. Their device comprised alternating sections of standard single-mode fiber (SMF) and polyimide-coated fiber, each exhibiting distinct sensitivities to environmental factors. The coated segment primarily responded to humidity variations due to the hygroscopic expansion of the polymer layer, whereas the uncoated section retained sensitivity, mainly to temperature fluctuations. By applying a trained data-driven regression model to the spectral dataset, the researchers achieved high measurement precision, with a root-mean-square error (RMSE) of 0.36 °C for temperature and 1.73% for relative humidity. This work illustrates how machine learning can significantly enhance the accuracy and practicality of hybrid OFDR multiparameter sensors, reducing the need for manual calibration and complex signal post-processing.

In [77], an optical frequency-domain reflectometry (OFDR) system capable of simultaneously measuring temperature, strain, and relative humidity was proposed and experimentally demonstrated. The authors utilized the fact that the Rayleigh backscattering (RBS) spectral shifts induced by temperature, strain, and humidity vary differently across the optical wavelength range. To exploit this property, the fiber-optic signal was divided into three distinct wavelength subregions, each analyzed separately. For every subregion, a cross-correlation operation was performed between the reference and measurement signals, allowing for the identification of spectral variations produced by individual environmental factors. The resulting wavelength-resolved spectrograms were used to construct a sensitivity matrix, which enabled accurate decoupling of the effects of temperature, strain, and humidity. In this way, the system was able to determine simultaneous changes in all three parameters without the need for additional equipment or specialized sensing fibers. The researchers validated their approach experimentally by inducing controlled, concurrent variations in temperature, humidity, and strain in the same optical fiber. Their results demonstrated that the proposed method provides high measurement accuracy even under strong mutual coupling between the measured parameters. According to the authors, this development offers several key advantages over existing multiparameter OFDR techniques. These include high sensitivity to environmental variations, straightforward integration with existing OFDR systems without the need for major hardware modifications, and broad application potential in fields such as engineering, biology, medicine, and environmental science, where multichannel parameter monitoring is increasingly in demand. However, the approach also presents certain limitations. The system requires a broadly tunable laser source, as wide-range wavelength scanning is necessary for proper operation. This tuning process inevitably reduces system speed, limiting performance in dynamic measurement scenarios. Moreover, the frequency-domain nature of the method brings both advantages and drawbacks. On one hand, only the frequency tuning of the radiation that is already a standard feature in OFDR systems is required to implement the technique. On the other hand, dividing the optical frequency range into multiple subranges leads to a reduction in spatial resolution. The more independent parameters are measured, the lower the reliability of spatial localization becomes.

Nevertheless, in principle, it is possible to envision arbitrarily fine segmentation of the frequency tuning range, thereby extending the number of measurable parameters without a fundamental redesign of the system. This scalability distinguishes the approach from most other multiparameter discrimination techniques, which generally do not allow for the addition of new measurable quantities without major structural modification. It is reasonable to assume that the strategic combination of this spectral-separation method with other techniques could mitigate its inherent drawbacks. In particular, approaches based on the polarization modes of anisotropic optical fibers and machine-learning or neural-network algorithms for feature extraction and parameter discrimination appear especially promising. Such hybrid architectures could retain the advantages of spectral-domain sensing while improving the accuracy, spatial resolution, and adaptability of OFDR-based multiparameter measurement systems.

The study presented in [78] offers an innovative machine-learning-based approach to analyzing the behavior of fiber-optic sensors, which is fundamentally different from traditional model-driven methods. Instead of relying on explicit analytical relationships, such as those described by Equations (30) and (31), the authors adopted a data-driven strategy, motivated by the practical difficulty of formulating strict analytical dependencies between the photodetector output signals and external environmental conditions such as temperature T and mechanical stress σ. In this work, machine learning (ML) techniques were employed to overcome these limitations, providing a flexible alternative to complex physical modeling. The researchers conducted controlled experiments in which an optical fiber was subjected to a matrix of environmental conditions comprising four discrete temperature levels and five levels of mechanical stress. These experiments yielded a substantial dataset of approximately 2000 unique backscattered signal samples collected from different fiber segments. The dataset was then used to train a neural network capable of identifying and quantifying the nonlinear correlations between the optical response and the applied physical stimuli. The trained model demonstrated high discrimination accuracy between temperature- and strain-induced effects. The temperature estimation error was below 2 K, while the mechanical stress error did not exceed 100 µε, confirming the strong potential of the ML-based approach for structural health monitoring and distributed condition assessment. Beyond the impressive quantitative results, the authors emphasized the broader conceptual significance of interpretable artificial intelligence (AI) in photonic sensing. They noted that conventional neural network models often operate as “black boxes,” providing accurate predictions without offering insight into their internal reasoning. To address this limitation, the authors proposed the development of interpretable AI frameworks, algorithms capable of visually or analytically highlighting the key spectral and temporal features that influence decision-making. Such transparency would not only enhance user trust but also allow experts to understand the dominant factors affecting signal classification and improve sensor calibration and design. This study thus represents a major step toward integrating machine learning and explainable AI into fiber-optic sensing, demonstrating that data-driven techniques can achieve both high precision and greater interpretability in the simultaneous discrimination of multiple environmental parameters.

#### 4.3.3. OFDR-Based Hybrid Systems

**Figure 16:**
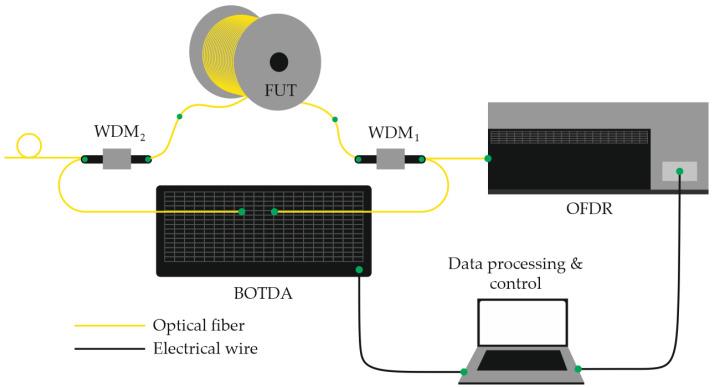
Schematic diagram of the experimental setup proposed by Zhou et al. for simultaneous monitoring of temperature and strain using combined RBS and Brillouin spectrum shifts. WDM—Wavelength Division Multiplexer.

Another promising direction in multiparameter sensing research involves the simultaneous use of Rayleigh and Brillouin scattering measurements, allowing for the combined extraction of complementary information from both effects [79]. The core concept of this hybrid approach is to monitor two independent frequency shifts: the Rayleigh backscattering (RBS) spectral shift Δν_1_ and the Brillouin scattering frequency shift Δν_2_. The configuration implementing this principle is schematically illustrated in Figure 16 of the referenced study.

In this setup, the two measurement subsystems—OFDR (based on RBS) and BOTDA (Brillouin optical time-domain analysis)—are optically linked via a 1310/1550 nm wavelength-division multiplexer (WDM1). A second multiplexer (WDM2) is used to extract the tunable laser source (TLS) beam from the fiber under test (FUT) after it traverses the sensing path. This design prevents distortion of the RBS signal caused by back-reflections within the BOTDA subsystem, such as those originating from the attenuator or optical isolator. To further minimize Fresnel reflections, a small fiber loop is formed at the output of the 1550 nm port of WDM2. During operation, the BOTDA subsystem is activated first to acquire Brillouin spectral data, after which the OFDR subsystem is initiated once the BOTDA sources are placed in standby mode. The two independent frequency shifts, Δν_1_ and Δν_2_, are subsequently converted into temperature *T* and strain σ values using calibration relationships analogous to Equations (30) and (31). The experimental system achieved a temperature measurement accuracy of approximately 1.2 K and a strain resolution of about 15 µε, with a sensing length of 92 m and a spatial resolution of 50 cm. This hybrid Rayleigh-Brillouin configuration thus provides a practical and efficient framework for the simultaneous distributed measurement of temperature and mechanical strain using a single optical fiber platform.

#### 4.3.4. An Example of the Multiparameter OFDR’s Application

Another important contribution in this field was made by Meng et al. [80], who addressed the temperature-induced distortions affecting the accuracy of strain-based shape reconstruction when using multi-core fibers (MCFs) in combination with optical frequency-domain reflectometry (OFDR). It is well known that temperature variations can significantly influence the quality of distributed strain data, leading to systematic errors in the determination of material properties and geometric parameters. To overcome this limitation, the authors proposed a configuration in which multiple central fiber cores operate in parallel, each acquiring nearly synchronous distributed measurements. This design enables the real-time compensation of temperature effects and improves the accuracy of the reconstructed structural geometry. In the initial stage, two outer cores of the MCF were used for three-dimensional shape reconstruction, employing vector projection algorithms to calculate the spatial deformation profile. After calibration, the system achieved a maximum relative reconstruction error of only 3.37%, confirming its ability to accurately reproduce the true shape of the tested object. In the subsequent phase, the researchers incorporated data from the central core of the MCF, allowing for the simultaneous monitoring of both temperature and fiber geometry. Experimental validation demonstrated stable system performance across a temperature range of 40–90 °C, with only a slight increase in the maximum relative error to 5.1%. These results underscore the high precision and robustness of the proposed approach for temperature-compensated shape sensing, highlighting the potential of multi-core OFDR systems in applications requiring accurate three-dimensional reconstruction under variable thermal conditions.

#### 4.3.5. A Brief Summary on the Multiparameter OFDR Method

In summary, all of the studies discussed above make a substantial contribution to the advancement of data processing methodologies and material property diagnostics in distributed fiber-optic sensing. Collectively, they lay the groundwork for the development of more efficient tools for monitoring and analyzing the structural health of engineering systems, thereby improving the reliability and accuracy of condition assessment technologies. Furthermore, the integration of machine-learning and artificial neural network approaches not only enhances data interpretation but also provides deeper insight into the internal mechanisms of AI-driven sensing systems. These developments significantly broaden the capabilities of modern structural monitoring and expand the horizons of intelligent engineering diagnostics. The optical frequency-domain reflectometry (OFDR) technique remains one of the most powerful methods for the precise measurement of temperature distributions and static or dynamic strain fields along optical fibers. Nevertheless, its practical implementation is often limited by high cost, largely due to the need for a tunable laser source with a wide and stable scanning range. This limitation can be mitigated by employing the Brillouin optical time-domain reflectometry (BOTDR) technique, which maintains sensitivity to both thermal and mechanical influences while enabling wavelength tuning through an electro-optic modulator rather than an expensive tunable laser. The principles, configurations, and results of simultaneous multiparameter measurements using BOTDR are discussed in detail in the next section.

## 5. Simultaneous Measurement of Several Physical Quantities Using BOTDR or BOTDA

### 5.1. Brillouin Scattering Basics and BFS—T-σ Sensitivity

In addition to distributed fiber-optic sensors based on Rayleigh scattering, those employing Brillouin scattering have also found wide application in monitoring temperature *T* and strain *σ* fields. Their principal advantage lies in the fact that they do not require a wavelength-tunable coherent radiation source with a wide tuning range (typically tens of nanometers). This feature is particularly valuable in applications such as aerospace structural monitoring, where measurements must often be performed through pre-installed onboard fiber networks. The use of self-injection-locked lasers offers a means to drastically simplify the classical Brillouin sensor architecture based on electro-optical modulators, reducing both system complexity and cost while maintaining high spectral purity and stability [81]. Furthermore, recent studies have demonstrated that even a simple, low-cost fiber laser with self-frequency scanning can be effectively employed for distributed Brillouin sensing, achieving reliable temperature and strain measurements with excellent repeatability [82].

Brillouin scattering arises from the interaction of an optical wave with acoustic vibrations (phonons) in the medium. This process is inelastic, as it involves either the absorption or emission of a phonon. When a phonon is absorbed, the optical wave gains energy, resulting in a frequency increase (the anti-Stokes component); conversely, when a phonon is emitted, the optical wave loses energy, producing the Stokes component. The frequency shift between the incident and scattered light, known as the Brillouin frequency shift $\nu_{b}$ is given by

(32) $$\nu_{b}=\frac{2n\upsilon }{\lambda }$$

where *n* is the refractive index of the fiber core, υ is the longitudinal acoustic velocity, and λ is the wavelength of light in vacuum.

**Figure 17:**
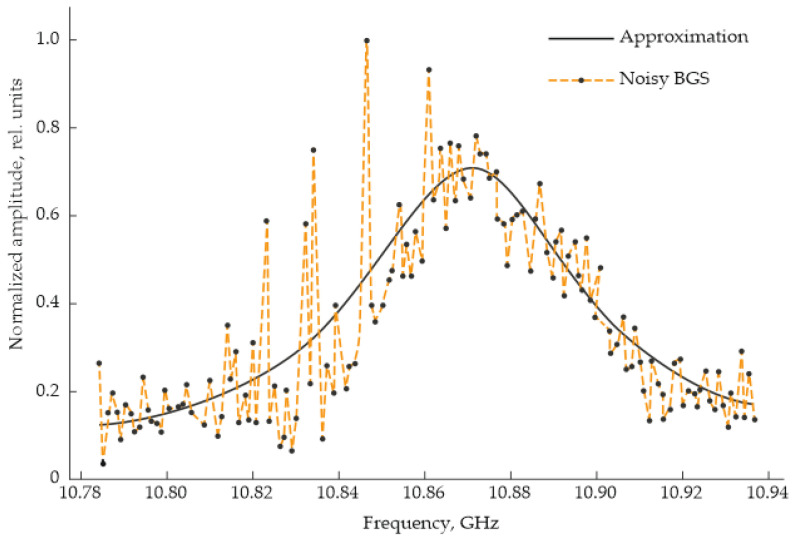
Idealized (approximated) and real (noisy) Brillouin Gain Spectrum (BGS) profile.

Two types of Brillouin scattering are distinguished: spontaneous and stimulated. Spontaneous Brillouin scattering forms the basis of Brillouin optical time-domain reflectometry (BOTDR). In this method, a single probing light wave is injected into the fiber, and the frequency of the backscattered radiation is measured along its length. As the BOTDR setup is largely similar to that of Φ-OTDR, except for the higher intensity of the laser source, Figure 7 provides a suitable reference for a basic BOTDR setup. The typical output data for a certain point along the fiber are presented in Figure 17.

Stimulated Brillouin scattering, on the other hand, occurs when acoustic phonons are coherently generated through electrostriction, forming the operating principle of Brillouin optical time-domain analysis (BOTDA). During this interaction, a narrowband pump wave propagates forward along the fiber and couples with a counterpropagating Stokes wave, whose frequency is downshifted relative to the pump by the Brillouin frequency shift (e.g., ~11 GHz at a wavelength of ~1550 nm). The resulting moving interference pattern excites resonant acoustic wave in the medium via the electrostriction effect. These acoustic waves cause periodic variations in the material density and refractive index, which boost optical backscattering. As a result, a positive feedback loop forms, leading to a resonant transfer of energy from the pump to the Stokes component [83]. In BOTDA, a probe wave is launched from one end of the fiber, while a pump wave with a slightly higher frequency is launched from the opposite end. To obtain the spatial distribution of the parameters under study, one of the two waves must be continuous, while the other is pulsed.

Two operational configurations are commonly employed. In Variant I, the probe wave is continuous, and the Brillouin amplification spectrum (BAS) is measured as the frequency-dependent amplification of the probe at the fiber output. In Variant II, the pump wave is continuous, and the Brillouin absorption spectrum (BAS) is measured as the frequency-dependent decrease in pump power. In practice, Variant II is generally preferred because it concentrates the majority of optical power in the pump wave, improving signal-to-noise ratio and measurement stability.

The evolution of optical powers along the fiber can be described by the following coupled equations:

(33) $$\left(\frac{1}{\upsilon_{g}}\frac{\partial }{\partial t}+\frac{\partial }{\partial z}+\alpha \right)P_{p}=-g(\nu )P_{p}P_{s},$$

(34) $$\left(\frac{1}{\upsilon_{g}}\frac{\partial }{\partial t}-\frac{\partial }{\partial z}+\alpha \right)P_{s}=g(\nu )P_{p}P_{s},$$

where *P_s_* and *P_p_* are the powers of the probe and pump waves, respectively; α is the fiber attenuation coefficient; *υ_g_* is the group velocity of light in the fiber; and z is the distance along the fiber axis.

The function g(ν) represents the Brillouin gain spectrum (BGS), which characterizes the dependence of gain on the frequency detuning between the two optical waves:

(35) $$g(\nu )=g_{\max}\frac{{\left(\frac{\Delta v}{2}\right)}^{2}}{{\left(\nu -\nu_{0}-\nu_{b}\right)}^{2}+{\left(\frac{\Delta \nu }{2}\right)}^{2}},$$

where ν and ν_0_ are the optical frequencies of the pump and probe waves, respectively, $\nu_{b}$ is the Brillouin frequency shift given by Equation (32), g_max_ is the peak Brillouin gain, and Δ*ν* is the full width at half maximum (FWHM) of the Brillouin gain spectrum.

A similar Lorentzian dependence also describes the Brillouin absorption spectrum, which is observed in the complementary configuration of the BOTDA system.

After a radiation pulse, either a pump or probe pulse, depending on the measurement configuration (Variant I or Variant II), is launched into the optical fiber, and the time-dependent backscattered power is recorded by a photodetector. The observed power variation (an increase in Variant I and a decrease in Variant II) reflects the efficiency of optical interaction within the region of the fiber where the pump and probe waves overlap. Thus, the Brillouin-based technique is conceptually analogous to classical OTDR, with the key distinction that the scattering centers are not static density fluctuations in the glass matrix, but rather acoustic phonons generated dynamically by the pump wave.

By scanning the frequency of the pulsed wave, the complete Brillouin amplification or absorption spectrum can be obtained for each fiber segment. Strictly speaking, the spatial resolution is determined not by a single point but by a section of fiber whose length corresponds to the pulse duration, which is again directly analogous to the OTDR operation. Because the scattering cross-section for Brillouin interactions—even in the stimulated regime—is much smaller than that for Rayleigh scattering, the resulting backscattered signal is extremely weak and the measured spectra are typically highly noisy (Figure 17). Identifying the Brillouin peak frequency through simple maximum detection introduces substantial errors; therefore, more sophisticated methods are employed in practice, including curve fitting and spectral approximation [84], cross-correlation analysis [85], and increasingly, machine-learning-based spectral interpretation [86]. Since both the refractive index *n* and the acoustic velocity *v* depend on temperature *T* and strain *σ*, the Brillouin frequency shift given by Equation (32) can be more accurately expressed as

(36) $$\nu_{b}=F(T,\sigma )$$

Because of this dual dependence, the Brillouin gain spectrum (BGS) cannot be directly used to extract temperature or strain independently. To achieve quantitative separation of these effects, multiparameter discrimination methods (as described in Section 2) must be applied. In such cases, the second measurable parameter could be another BGS (for example, obtained using a fiber of a different type or a distinct optical/acoustic mode of the same fiber) or an additional Brillouin-related quantity, such as the scattering power or spectral linewidth. A brief overview of recent studies presenting modern approaches for the simultaneous measurement of temperature *T* and strain *σ* in optical fibers using Brillouin-based analysis and BOTDR techniques is provided below.

### 5.2. T-σ Separation Strategies in BOTDA (Hardware and Signal Design)

Because BOTDA relies on stimulated Brillouin scattering with dual-ended access, it enables the active shaping of the BGS and a significantly higher signal-to-noise ratio. This makes it a favorable platform for implementing hardware-assisted temperature-strain discrimination.

In [87], Sheng et al. proposed a multiparameter distributed fiber-optic sensor for the simultaneous monitoring of temperature and strain fields based on spontaneous Brillouin scattering in a polyimide-coated optical fiber. The authors achieved effective discrimination between *T* and *σ* by analyzing the sensitivity coefficients of Brillouin frequency shifts corresponding to different acoustic modes. It was demonstrated that these modes exhibit sufficiently distinct sensitivity coefficients, enabling accurate parameter separation. By tracking the Brillouin frequency shifts in two dominant spectral peaks, the system successfully measured strain and temperature simultaneously with accuracies of 19.68 µε and 1.02 °C, respectively, over a 2.5 km long sensing fiber. The method thus provides reliable performance for practical applications such as oil and gas exploration, where distributed and precise *T-σ* discrimination is essential.

In [88], Murray et al. developed a hybrid distributed sensor combining a time-domain Brillouin analyzer with a wavelength-scanning Rayleigh reflectometer. This configuration is conceptually similar to previously discussed hybrid schemes in which the discrimination of *T* and *σ* was achieved using two complementary systems; however, in this case, the Rayleigh subsystem operates in the wavelength (frequency) domain, while the Brillouin subsystem operates in the time domain. The hybrid sensor demonstrated the ability to separate thermal effects from both dynamic and quasi-static strain variations with high precision. The achieved measurement resolutions were 16 mK for temperature and 140 nε for strain, along a 500 m single-mode fiber with a spatial resolution of 5 m. Moreover, the system enabled dynamic measurements with a 1.7 kHz bandwidth and spectral noise densities of 0.54 m°C/√Hz for temperature and 4.5 nε/√Hz for strain. Importantly, temperature-strain cross-sensitivity was suppressed by more than 25 dB, confirming the robustness of the method. Overall, the approach presented by Murray et al. not only ensures high discrimination accuracy between *T* and *σ* but also achieves rapid measurement speed, making it suitable for real-time distributed sensing in demanding environments such as industrial process monitoring and structural diagnostics.

In [79], Zhou et al. demonstrated a hybrid distributed fiber-optic sensor capable of simultaneously measuring strain *σ* and temperature *T*, employing a large-effective-area, non-zero-dispersion optical fiber with sub-meter spatial resolution. In this system, the Brillouin frequency shift was obtained using optical Brillouin time analysis (BOTDA) based on the differential pulse-width technique, while the Rayleigh backscattering spectrum shift was determined through optical frequency-domain reflectometry (OFDR). Since both spectral shifts are functions of *σ* and *T*, they serve as two independent observables, enabling precise discrimination of these physical quantities. Experimental validation demonstrated reliable performance over a 92 m fiber segment with a spatial resolution of 50 cm. The achieved accuracies were ±1.2 °C for temperature and ±15 µε for strain. This hybrid configuration effectively combines the high spatial precision of OFDR with the broad dynamic range of Brillouin-based techniques, offering a practical approach for applications requiring both fine resolution and multiparameter capability.

In [89], Sabatier et al. presented a prototype distributed fiber-optic sensor designed for the simultaneous measurement of temperature and strain under high-radiation environments. The system combines the Landau-Placzek ratio with the Brillouin frequency shift to separate the effects of multiple physical quantities along optical fibers up to 10 km in length, with a spatial resolution of 1 m. A key feature of this work is its focus on radiation-tolerant sensing. The authors demonstrated that the Landau-Placzek ratio remains stable even under radiation doses up to the megagray (MGy) level, whereas the Brillouin spectral frequency experiences only minor shifts under similar conditions. Furthermore, it was shown that using fluorine-doped single-mode fibers—known for their enhanced radiation resistance—significantly reduces Brillouin frequency shifts caused by ionizing radiation. This finding confirms that such fibers can be effectively integrated into distributed Brillouin-based sensing systems, enabling simultaneous temperature and strain measurement even in radiation-contaminated environments such as nuclear facilities, spacecraft, and high-energy physics installations.

In [90], Li et al. demonstrated a multiparameter distributed fiber-optic sensor based on a low-mode fiber (LMF) for the simultaneous measurement of strain σ and temperature T. In their approach, the pump and probe signals were launched into the linearly polarized modes of the LMF, and the Brillouin frequency shifts corresponding to each mode were recorded. By analyzing the temperature and strain sensitivity coefficients of the two lowest-order modes (LP_01_ and LP_11_), the authors experimentally achieved clear discrimination between *T* and *σ*, with accuracies of 1.2 °C and 21.9 µε, respectively. This method is particularly attractive due to its conceptual simplicity and ease of implementation. However, the limited availability and practical adoption of low-mode fibers significantly constrains the widespread use of this technique in existing fiber infrastructures, where standard single-mode fibers dominate. It should also be noted that researchers are continuing to explore another well-established direction—the use of anisotropic, polarization-maintaining fibers (PMFs)—for *T*-*σ* discrimination. As discussed in [21], a model of polarization-Brillouin reflectometry was developed to evaluate the effect of instrumental and calibration errors on the accuracy of temperature and strain separation. This approach relies on measuring the Brillouin frequency shifts along the two orthogonal polarization axes of a PM fiber. Both simulation and experimental data confirmed that the precise determination of calibration coefficients for each polarization mode is critical for accurate parameter separation. The main limitation of this early method, however, was the need to sequentially measure the Brillouin frequency shifts in both polarization axes, requiring manual or automated switching of the fiber orientation. To overcome this, the same research group later proposed a technique for the simultaneous observation of frequency shifts along both polarization axes [91]. This was achieved by employing two polarization beam splitters properly aligned with a Brillouin analyzer and the fiber under test. The modified configuration enabled the acquisition of two Brillouin traces—one for each polarization axis—within a single frequency sweep. Nevertheless, the authors reported that despite the improved measurement efficiency, the complete and accurate separation of temperature and strain influences was not fully achieved within this setup.

### 5.3. Adaptation to BOTDR (Single-Ended Constraints, Denoising)

Many of the *T*-σ discrimination strategies originally developed for BOTDA can, in principle, be transferred to BOTDR; however, their practical implementation must be adapted to the fundamentally different operating conditions of spontaneous Brillouin scattering. Unlike BOTDA, which benefits from a counterpropagating pump-probe interaction that shapes the Brillouin gain spectrum and enhances the signal-to-noise ratio (SNR), BOTDR is a single-ended technique in which the detected Stokes component arises solely from thermally excited acoustic phonons. As a consequence, the backscattered power is several orders of magnitude lower, the Brillouin spectra are noisier, and the extracted Brillouin frequency shift (BFS) exhibits significantly higher uncertainty.

Because of these limitations, BOTDR systems require substantially longer averaging times and more advanced denoising procedures to recover the BFS with acceptable precision. Classical curve-fitting approaches (Lorentzian or Voigt approximations [84]), cross-correlation spectral alignment [85], and more sophisticated machine-learning-based spectral reconstruction and peak tracking [86] are routinely employed to compensate for the reduced SNR. In addition to the BFS, BOTDR analysis often incorporates auxiliary spectral descriptors—such as Brillouin amplitude, linewidth, or spectral asymmetry—because these parameters help stabilize the inversion matrix required for *T*-*σ* separation.

Several BOTDA concepts can nevertheless be adapted to BOTDR when sufficient SNR is available. Multi-acoustic-mode discrimination, as demonstrated in [87], remains feasible under spontaneous scattering, although higher-order modes may require increased acquisition time or engineered fibers. Similarly, the use of distinct fiber modes (LP_01_/LP_11_) in low-mode fibers [90] is theoretically compatible with BOTDR, but the inherently weaker scattering efficiency in higher-order modes poses additional challenges. Hybrid spectral schemes that exploit Rayleigh-Brillouin diversity—such as OFDR-BOTDA combinations [79]—can also be translated into BOTDR-OFDR architectures, provided that the Rayleigh subsystem supplies a complementary high-SNR observable.

Radiation-tolerant discrimination based on the Landau-Placzek ratio [89] is particularly well suited to BOTDR, because this ratio is defined entirely through spontaneous Rayleigh and Brillouin components and therefore remains measurable without a stimulated interaction. This makes the approach attractive in harsh environments—such as nuclear setups or space systems—where pump amplification is impractical or undesirable.

While the hardware measures discussed earlier were introduced in the context of BOTDA, several underlying strategies—multi-wavelength or modal diversity, polarization-resolved detection, dual-parameter regression, and machine-learning-assisted demodulation—are equally applicable to BOTDR. However, their implementation differs substantially because BOTDR relies on spontaneous scattering, operates with single-ended access, and therefore exhibits a significantly lower SNR. As a result, BOTDR requires longer integration times, more aggressive denoising (curve-fitting, cross-correlation tracking, ML-based spectral reconstruction), and often exploits additional spectral features—such as Brillouin amplitude or linewidth—to stabilize the *T*-*σ* inversion. Unlike BOTDA, BOTDR cannot employ a counter-propagating pump to control the Brillouin gain, meaning that discrimination performance depends more strongly on fiber type, acoustic modes, and optimized probe-pulse design.

Thus, while many conceptual *T*-*σ* discrimination approaches are transferable from BOTDA to BOTDR, their practical realization must be carefully adapted to the lower optical power, noisier spectra, and single-ended geometry inherent to BOTDR systems.

### 5.4. Hybrid Rayleigh-Brillouin Methods for Multiparameter Sensing

Hybrid interrogation schemes that combine Rayleigh and Brillouin scattering measurements have emerged as one of the most effective strategies for improving *T*-*σ* discrimination in distributed sensing. These methods exploit the complementary physical sensitivities of the two scattering processes: Rayleigh backscatter primarily tracks refractive-index and structural changes, while the Brillouin response additionally depends on acoustic velocity and therefore provides an independent *T*-*σ* observable.

Several hybrid system architectures have demonstrated significant improvements in accuracy, spatial resolution, and dynamic range. In [88], a Rayleigh reflectometer operating in the wavelength-scanning regime was combined with a time-domain Brillouin analyzer. This dual-domain approach enabled simultaneous, high-speed discrimination between thermal effects and dynamic or quasi-static strain variations, achieving *T* and *σ* resolutions of 16 mK and 140 nε, respectively, with a 1.7 kHz dynamic bandwidth. The ability to suppress *T*-*σ* cross-sensitivity by more than 25 dB highlights the advantage of combining spectral diversity (Rayleigh subsystem) with Brillouin gain-shift sensitivity (Brillouin subsystem).

In [79], Zhou et al. combined OFDR-based Rayleigh spectral-shift interrogation with BOTDA measurements in a large-effective-area fiber. This configuration provided two independent observables for *T*-*σ* separation, enabling sub-meter spatial resolution (50 cm) and accurate discrimination with ±1.2 °C and ±15 µε precision. This hybrid OFDR-Brillouin sensing approach is particularly attractive because it unites the ultra-high spatial resolution of OFDR with the long-range mapping capabilities of Brillouin analysis.

Radiation-tolerant hybrid sensing was demonstrated in [89], where the Landau-Placzek ratio (derived from spontaneous Rayleigh and Brillouin components) was combined with BFS measurements to achieve robust multi-parameter monitoring in harsh radiation environments. The system remained stable under MGy-level gamma irradiation, especially when fluorine-doped fibers were used to mitigate radiation-induced changes in Brillouin spectra.

In another hybrid configuration, Wang et al. [92] integrated dynamic Brillouin gratings (DBGs) with Rayleigh backscattering in polarization-maintaining fibers. Due to the strong birefringence of PM fibers, temperature and strain variations induce opposite-sign spectral shifts in DBG responses, enabling highly accurate discrimination. Using a single-pulse chirped interrogation, the authors achieved errors of only 112.2 nε and 10.9 m°C at 2 m spatial resolution—one of the most precise demonstrations of Rayleigh-Brillouin hybrid sensing to date.

Finally, in [93], a hybrid Brillouin-Rayleigh system was implemented using wavelength-division multiplexing (WDM), with deep neural networks employed for denoising and event classification. This approach enabled the simultaneous measurement of static strain, dynamic strain, and temperature over 25 km with a spatial resolution of 3 m, illustrating the potential of combining optical multiplexing with advanced computational tools for next-generation distributed sensing.

Together, these works demonstrate that hybrid Rayleigh-Brillouin techniques provide a powerful platform for achieving high-accuracy multiparameter interrogation, particularly in applications requiring a long range, high resolution, or environmental robustness.

### 5.5. Machine Learning for BGS Fitting and Multiparameter Inversion

The intrinsic weakness of Brillouin scattering—especially in BOTDR systems—makes the extraction of the Brillouin frequency shift (BFS), linewidth, and amplitude highly susceptible to noise. As a result, machine-learning (ML) methods have increasingly become an essential part of the data-processing toolbox for distributed Brillouin sensing.

Traditional denoising and spectral-fitting methods include Lorentzian curve-fitting [84], cross-correlation peak-tracking [85], and multi-parameter spectral approximation. However, ML approaches can outperform analytical models when spectra are heavily distorted, when multiple parameters overlap, or when the physical model is difficult to invert directly. For example, neural-network-based BGS reconstruction has been shown to robustly estimate BFS even under extremely low SNR conditions [86].

In [69], a supervised learning model was trained on approximately two thousand experimentally collected Brillouin spectra, each corresponding to different combinations of *T* and *σ*. The trained network achieved prediction errors below 2 K and 100 µε without requiring explicit analytical modeling of the Brillouin gain spectrum. This approach highlights the potential of ML to bypass complex inversion models and directly learn parameter mappings from empirical data.

The most advanced integration of ML into hybrid sensing systems was demonstrated in [93], where deep neural networks were incorporated into a combined Rayleigh-Brillouin WDM system. The DNN architecture performed denoising, reconstruction, and event classification simultaneously, significantly improving BFS extraction and enabling precise detection of static and dynamic perturbations over 25 km.

Overall, machine-learning-enhanced Brillouin sensing is particularly valuable for BOTDR (low-SNR conditions), for multi-parameter discrimination, and for real-time dynamic monitoring where conventional fitting methods are computationally prohibitive.

### 5.6. Practical Considerations: Dynamic Range, Spatial Resolution, Calibration

The practical deployment of distributed Brillouin sensing systems requires careful attention to several engineering constraints—including spatial resolution, dynamic range, SNR, and calibration.

Spatial resolution in BOTDA and BOTDR is fundamentally limited by pulse width: typical systems achieve 0.5–2 m resolution, while sophisticated differential pulse-width methods can achieve tens of centimeters. Hybrid BOTDA-OFDR architectures [79] can reach sub-meter resolution by leveraging the ultra-fine Rayleigh spectral-shift sensitivity of OFDR.

Dynamic measurement capability varies substantially across systems. BOTDA systems optimized for high-speed operation can achieve kHz-level dynamic bandwidths (e.g., 1.7 kHz in [88]), enabling the real-time monitoring of vibrations and dynamic strain. BOTDR systems, due to their inherently lower SNR, generally require longer averaging times and are therefore more suitable for quasi-static or slowly varying fields unless enhanced by ML or signal processing.

Environmental conditions also impose important practical constraints. Radiation-induced changes in fiber characteristics, such as Radiation-Induced Attenuation (RIA) and BFS drift, can significantly degrade sensing accuracy. As shown in [89], fluorine-doped fibers greatly reduce BFS under MGy exposure, making them ideal for nuclear or space applications. The Landau-Placzek ratio further enhances discrimination stability under radiation.

Accurate parameter separation requires careful calibration of the *T* and *σ* sensitivity coefficients for each fiber type—standard SMF, polyimide-coated fibers [87], PM fibers [21,91], and low-mode fibers [90]—as well as for specific acoustic or optical modes. Variations caused by microbending, local heating, or diameter fluctuations must be compensated, particularly in field-deployed systems.

In summary, achieving robust and accurate multiparameter Brillouin sensing requires a coordinated optimization strategy encompassing appropriate fiber selection for the target environment; a pulse-width and interrogation-scheme design for the desired resolution; SNR enhancement and denoising (including ML-based methods); calibration of BFS sensitivity coefficients; and compensation for environmental or radiation-induced perturbations. When these considerations are addressed, BOTDA/BOTDR and hybrid Brillouin-Rayleigh systems can deliver highly reliable and precise distributed *T*-*σ* measurements over tens of kilometers.

## 6. Conclusions

This review summarized both the state-of-the-art and classical approaches to multiparameter measurements using distributed fiber-optic sensors. All of the optical mechanisms considered—Rayleigh, Brillouin, and Raman scattering—are inherently multiparametric, since several external quantities such as temperature, strain, pressure, and acoustic vibrations simultaneously influence the recorded optical response. Thus, the primary challenge is not sensing itself but the accurate registration, separation, and interpretation of overlapping physical effects. When appropriately designed, distributed fiber-optic sensors provide a powerful and highly informative platform capable of delivering spatially resolved measurements of multiple environmental and structural parameters along a single optical fiber.

The sensitivity of Rayleigh and Brillouin scattering spectra to both temperature and strain has enabled multiparameter sensing using optical frequency-domain reflectometry (OFDR) and Brillouin optical time-domain reflectometry (BOTDR), respectively. However, reliable discrimination of these effects requires additional strategies. One established solution is the use of polarization-maintaining fibers, whose orthogonal polarization axes exhibit different thermo-mechanical sensitivities. When implemented on modern optoelectronic components and enhanced with interpretable machine-learning algorithms, this method provides substantial improvements in temperature-strain discrimination. Another widely used strategy is spectral diversity, in which a broad laser-tuning range is subdivided into narrow sub-bands, each with distinct parameter sensitivities. This enables the discrimination of several simultaneously acting quantities, albeit at the cost of reduced spatial resolution within each sub-band. In Brillouin systems, the use of different acoustic modes offers an alternative pathway without requiring anisotropic fibers. In Rayleigh-based systems such as COTDR and Φ-OTDR, hybrid Rayleigh-Raman schemes and the temporal separation of slow (*T*, *σ*) and fast (acoustic) effects provide additional routes to multiparameter sensing.

A major recent trend is the integration of artificial intelligence (AI) and neural networks into the processing pipeline. In many cases, researchers bypass explicit analytical models and directly train neural networks on experimentally obtained spectral data influenced by multiple parameters. This approach can significantly improve discrimination accuracy; however, “black-box” models may obscure the underlying physics, motivating the parallel development of interpretable and physics-informed AI.

In addition to laboratory prototypes, several commercial distributed sensing systems are already available, demonstrating that many of the technologies reviewed here have reached a stage of practical maturity. Rayleigh-based OFDR/HD-FOS instruments such as the Luna ODiSI series (and OBR reflectometers) offer sub-millimeter to centimeter spatial resolution for high-definition temperature and strain mapping. For acoustic sensing, Silixa iDAS and OptaSense interrogators represent mature Φ-OTDR-class products operating over multi-kilometer distances with a high dynamic range. In the Brillouin domain, BOTDA/BOTDR platforms from companies such as Omnisens (DiTeSt/ViSION) and OZ Optics (ForeSight DSTS) deliver long-range temperature-strain mapping over tens of kilometers. These commercial instruments generally provide stability, ruggedness, and integrated calibration, while research prototypes often explore more advanced discrimination mechanisms at the cost of complexity.

Based on current trends, we expect that hybrid (Rayleigh + Brillouin) sensors incorporating polarization or modal diversity and interpretable ML will achieve, within the next 3–5 years, simultaneous strain noise floors below ~20–50 nε/√Hz and temperature precision better than 0.05–0.1 K at meter-scale resolution over 10–20 km. For short-range OFDR-class systems, static strain precision on the order of 1–5 µε and temperature accuracy near 10–30 mK at centimeter-scale resolutions appear realistic. Over longer time horizons, further improvements are anticipated as multi-modal data fusion and 2D/3D spectral analysis (using full spectral surfaces rather than only peak shifts) become standard practice.

**Figure 18:**
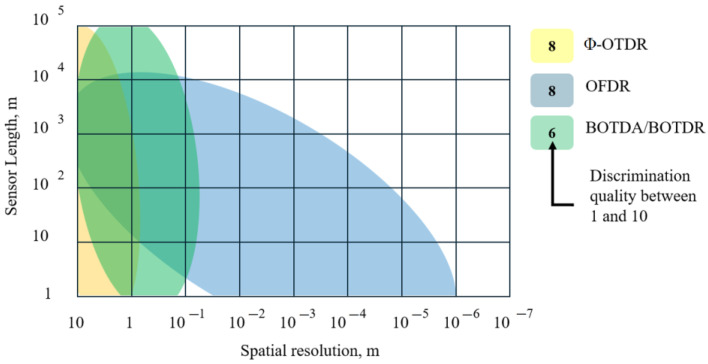
Three regions describing combinations of spatial resolution and fiber optic sensor length, rated using a ten-point scale based on the reviewed literature. Yellow region—Φ-OTDR [48,49,50,51,52,53,54,55,56,57,58,59,60,61], blue—OFDR [71,72,73,74,75,76,77,78,79], green—BOTDA/BOTDR [87,88,89,90].

A broader analysis of the reviewed literature indicates that the three principal distributed sensing modalities—Rayleigh, Brillouin, and hybrid systems—are evolving toward increasing complementarity rather than competition. Long-term development is likely to focus on multimodal, hybrid, and data-adaptive architectures that explicitly target cross-sensitivity. Future high-performance systems will integrate multiple scattering mechanisms, multi-core fibers [94,95,96,97,98,99,100] or photonic-crystal fibers [101,102,103,104,105,106,107,108,109] with intelligent coatings [110,111,112,113,114], and adaptive AI that optimizes interrogation parameters in real time [115,116,117,118,119,120,121]. Because the achievable accuracies and ranges vary widely across methods, a strict quantitative comparison is not always practical; instead, Figure 18 summarizes the qualitative performance domains of representative approaches. As the amount and dimensionality of processed spectral data increase, using the full spectral response rather than peak values alone will unlock additional information currently unused in classical demodulation. Such progress may yield order-of-magnitude improvements in simultaneous multi-parameter accuracy, ultimately accelerating the transition of multiparameter distributed sensing from research laboratories into robust, commercial-grade instrumentation.

In conclusion, the continued co-development of optimized optical hardware, rigorous analytical modeling, and physically interpretable AI constitutes the most promising pathway toward the next generation of multiparameter distributed fiber-optic sensing technologies.

## Figures and Tables

**Table 1:** Comparison of the main achievements in the field of multiparameter measurements in Φ-OTDR systems.

Reference	Discrimination Type	Effects	Accuracy	Range	Spatial Resolution, m	Fiber Length, km
[48]	Wavelength division, software	Acoustic	–	500 Hz	5.0	5.000
		Thermal	0.5 °C	0, 25, 50 °C		
[49]	Time gating, software	Acoustic	–	–	2.0	33.000
		Thermal	–	52.8–50.3 °C		
[50]	Software	Acoustic	4 nε	1–850 Hz, −300–300 nε	10.0	1.000
		Thermal	1 mK	23–27.5 °C		
[51]	Software, wavelength division	Acoustic	–	Up to 4800 Hz	3	10.000
		Thermal	–	24.8–72.5 °C	0.8	
		Deforming	–	Up to 2000 με	0.8	
[52]	Spatial	Acoustic	–	16–81 °C	2.5	1.565
		Thermal	0.001 °C	–		
[53]	Spatial	Acoustic	–	Up to 6250 Hz	3.0	5.760
		Thermal	0.5 °C	50, 60, 75 °C		
[54]	Wavelength division	Acoustic	–	100–1000 Hz	10.0	12.000
		Thermal	0.95 °C	35–55 °C		
[55]	Software	Acoustic	–	From 5 Hz	10.0	55.000
		Thermal	0.038–0.4 °C	24.7–31.2 °C		
[56,57]	Spatial, wavelength division	Acoustic	–	200; 5000 Hz	10.0	4.000
		Thermal	1 °C	7.2, 22, 48.3 °C		
[58]	Spatial, software	Deforming (salinity)	0.0469 mol/L	–	–	–
		Thermal	0.0344 K	–		
[60]	Wavelength division, software	Acoustic	–	–	6.0	12.000
		Thermal	0.85 °C	−0.7–1.3 °C		
[61]	Time gating	Acoustic	–	up to 0.6 Hz	–	–
		Thermal	–	–		

## Data Availability

Not applicable.

## Abbreviations

The following abbreviations are used in this manuscript:

AI	Artificial intelligence
AOM	Acousto-optical modulator
APD	Avalanche photodetector
BAS	Brillouin absorption spectrum
BGS	Brillouin gain spectrum
BL	Brillouin laser
BPF	Band-pass filter
BOTDA	Brillouin optical time-domain analyzer
BOTDR	Brillouin optical time-domain reflectometry
CCF	Cross-correlation function
C-OFDR	Coherent OFDR
COTDR	Coherent OTDR
DAS	Distributed acoustic sensor
DAQ	Data acquisition card
DFB	Distributed feedback
DNN	Deep neural network
DTS	Distributed temperature sensor
EDFA	Erbium-doped fiber optical amplifier
FMCW	Frequency-modulated continuous-wave
FUT	Fiber under test
FWHM	Full width at half-maximum
LD	Laser diode
LMF	Low-mode fiber
MCF	Multi-core fiber
ML	Machine learning
MMF	Multi-mode fiber
MZM	Mach-Zender Modulator
NL	Narrow-line
OFDR	Optical frequency-domain reflectometry
OS	Optical switch
OSA	Optical spectrum analyzer
OTDR	Optical time-domain reflectometry
PC	Polarization controller
PD	Photodetector
PIN	p-i-n photodetector
PMF	Polarization maintaining fiber
PS	Polarization switch/polarization scrambler
PZT	Piezoelectric transducer
RBS	Rayleigh backscattering
RIA	Radiation-induced absorption
ROTDR	Raman optical time-domain reflectometry
SMF	Single-mode fiber
SNR	Signal-to-noise ratio
SOA	Semiconductor optical amplifier
TCC	Thermally controlled chamber
TLS	Tunable laser source
UWFBG	Ultra-weak fiber Bragg gratings
VOA	Variable optical attenuator
WDM	Wavelength division multiplexer
Φ-OTDR	Phase-sensitive OTDR

## References

[1] Taranov M.A., Gorshkov B.G., Alekseev A.E., Konstantinov Y.A., Turov A.T., Barkov F.L., Wang Z., Zhao Z., Zan M.S.D., Kolesnichenko E.V. (2023). Optical reflectometry, metrology, and sensing. present and future. Instrum. Exp. Tech..

[2] Alekseev A.E., Gorshkov B.G., Potapov V.T., Taranov M.A., Simikin D.E. (2023). A Fiber Phase-Sensitive Optical Time-Domain Reflectometer for Engineering Geology Application. Instrum. Exp. Tech..

[3] Gorshkov B.G., Simikin D.E., Alekseev A.E., Taranov M.A., Zhukov K.M., Potapov V.T. (2023). Brillouin-Scattering induced noise in DAS: A case study. Sensors.

[4] Matveenko V., Fedorov A., Serovaev G., Galkina E., Vindokurova E. (2025). Flange joint monitoring based on strain measurement with fiber optic sensors embedded in gaskets. Sens. Int..

[5] Matveenko V., Serovaev G. (2023). Distributed Strain Measurements Based on Rayleigh Scattering in the Presence of Fiber Bragg Gratings in an Optical Fiber. Photonics.

[6] Bogachkov I.V. (2023). Creation of Adaptive Algorithms for Determining the Brillouin Frequency Shift and Tension of Optical Fiber. Instrum. Exp. Tech..

[7] Nonogaki H., Sei D., Zan M.S.D., Tanaka Y. (2024). Brillouin frequency shift measurement by zero-crossing point search in virtually synthesized spectra of Brillouin gain and loss. Appl. Phys. Express.

[8] Zhan Y., Li K., Zhang W., Liu L., Han M., Wang Z., Yang J., Liu Y., Ye Q. (2024). The DAS With Deep Neural Network Based on DSR-Net for Fast Earthquake Recognition. IEEE Photonics J..

[9] Shen Z., Wu W. (2024). Ocean bottom distributed acoustic sensing for oceanic seismicity detection and seismic ocean thermometry. J. Geophys. Res. Solid Earth.

[10] Ashry I., Wang B., Mao Y., Sait M., Guo Y., Al-Fehaid Y., Al-Shawaf A., Ng T.K., Ooi B.S. (2022). CNN–aided optical fiber distributed acoustic sensing for early detection of red palm weevil: A field experiment. Sensors.

[11] Ashry I., Mao Y., Al-Fehaid Y., Al-Shawaf A., Al-Bagshi M., Al-Brahim S., Ng T.K., Ooi B.S. (2020). Early detection of red palm weevil using distributed optical sensor. Sci. Rep..

[12] Stepanov K.V., Zhirnov A.A., Sazonkin S.G., Pnev A.B., Bobrov A.N., Yagodnikov D.A. (2022). Non-invasive acoustic monitoring of gas turbine units by fiber optic sensors. Sensors.

[13] Belokrylov M.E., Kambur D.A., Konstantinov Y.A., Claude D., Barkov F.L. (2024). An optical frequency domain reflectometer’s (OFDR) performance improvement via empirical mode decomposition (EMD) and frequency filtration for smart sensing. Sensors.

[14] Cheng F. (2024). Photonic seismology: A new decade of distributed acoustic sensing in geophysics from 2012 to 2023. Surv. Geophys..

[15] Li J., Zhu W., Biondi E., Zhan Z. (2023). Earthquake focal mechanisms with distributed acoustic sensing. Nat. Commun..

[16] Zhao M., Guo Y., Zhan X., Wang T., Shen X. Study of cross-sensitivity in the fiber-optic sensor based on pervasive computing. Proceedings of the 2006 First International Symposium on Pervasive Computing and Applications.

[17] Qin H., Tang P., Lei J., Chen H., Luo B. (2023). Investigation of strain-temperature cross-sensitivity of FBG strain sensors embedded onto different substrates. Photonic Sens..

[18] Yuksel K., Wuilpart M., Moeyaert V., Mégret P. Optical frequency domain reflectometry: A review. Proceedings of the 2009 11th International Conference on Transparent Optical Networks.

[19] Liu S., Yu F., Hong R., Xu W., Shao L., Wang F. (2025). Advances in phase-sensitive optical time-domain reflectometry. Opto-Electron. Adv..

[20] Kurashima T., Horiguchi T., Izumita H., Furukawa S.I., Koyamada Y. (1993). Brillouin optical-fiber time domain reflectometry. IEICE Trans. Commun..

[21] Barkov F.L., Konstantinov Y.A., Burdin V.V., Krivosheev A.I. (2020). Theoretical and experimental estimation of the accuracy in simultaneous distributed measurements of temperatures and strains in anisotropic optical fibers using polarization-brillouin reflectometry. Instrum. Exp. Tech..

[22] Maurer R.D., Schultz P.C. (1972). Fused silica optical waveguide. U.S. Patent.

[23] Barnoski M.K., Jensen S.M. (1976). Fiber waveguides: A novel technique for investigating attenuation characteristics. Appl. Opt..

[24] Lu P., Lalam N., Badar M., Liu B., Chorpening B.T., Buric M.P., Ohodnicki P.R. (2019). Distributed optical fiber sensing: Review and perspective. Appl. Phys. Rev..

[25] Palmieri L., Schenato L. (2013). Distributed optical fiber sensing based on Rayleigh scattering. Open Opt. J..

[26] Healey P., Malyon D.J. (1982). OTDR in single-mode fibre at 1.5 μm using heterodyne detection. Electron. Lett..

[27] Han B., Guan H., Yao J., Rao Y.J., Ran Z., Gong Y., Li Q., Li M., Zhang R., An S. (2020). Distributed acoustic sensing with sensitivity-enhanced optical cable. IEEE Sens. J..

[28] Taylor H.F., Lee C.E. (1993). Apparatus and method for fiber optic intrusion sensing. U.S. Patent.

[29] Turov A.T., Konstantinov Y.A., Barkov F.L., Korobko D.A., Zolotovskii I.O., Lopez-Mercado C.A., Fotiadi A.A. (2023). Enhancing the distributed acoustic sensors’(das) performance by the simple noise reduction algorithms sequential application. Algorithms.

[30] Masoudi A., Belal M., Newson T.P. (2013). A distributed optical fibre dynamic strain sensor based on phase-OTDR. Meas. Sci. Technol..

[31] Dong Y., Chen X., Liu E., Fu C., Zhang H., Lu Z. (2016). Quantitative measurement of dynamic nanostrain based on a phase-sensitive optical time domain reflectometer. Appl. Opt..

[32] Hartog A., Frignet B., Mackie D., Clark M. (2014). Vertical seismic optical profiling on wireline logging cable. Geophysical Prospecting 2014, 62(4-Vertical Seismic Profiling and Microseismicity Frontiers).

[33] Parker T.R., Shatalin S.V., Farhadiroushan M., Miller D. Distributed acoustic sensing: Recent field data and performance validation. Proceedings of the Second EAGE Workshop on Permanent Reservoir Monitoring 2013–Current and Future Trends.

[34] Liu Q., Liu T., He T., Li H., Yan Z., Zhang L., Sun Q. (2021). High resolution and large sensing range liquid level measurement using phase-sensitive optic distributed sensor. Opt. Express.

[35] Ren L., Jiang T., Jia Z.G., Li D.S., Yuan C.L., Li H.N. (2018). Pipeline corrosion and leakage monitoring based on the distributed optical fiber sensing technology. Measurement.

[36] Peng Z., Jian J., Wen H., Gribok A., Wang M., Liu H., Huang S., Mao Z.H., Chen K.P. (2020). Distributed fiber sensor and machine learning data analytics for pipeline protection against extrinsic intrusions and intrinsic corrosions. Opt. Express.

[37] MacLean A., Moran C., Johnstone W., Culshaw B., Marsh D., Parker P. (2003). Detection of hydrocarbon fuel spills using a distributed fibre optic sensor. Sens. Actuators A Phys..

[38] Bakhoum E.G., Zhang C., Cheng M.H. (2020). Real time measurement of airplane flutter via distributed acoustic sensing. Aerospace.

[39] Chen M., Li B., Masoudi A., Bull D., Barton J.M. Distributed Optical Fibre Sensor for Strain Measurement of Reinforced Concrete Beams. Proceedings of the 2020 International Conference on Intelligent Transportation, Big Data & Smart City (ICITBS).

[40] Li Z., Zhang J., Wang M., Zhong Y., Peng F. (2020). Fiber distributed acoustic sensing using convolutional long short-term memory network: A field test on high-speed railway intrusion detection. Opt. Express.

[41] Wang B., Mao Y., Ashry I., Al-Fehaid Y., Al-Shawaf A., Ng T.K., Yu C., Ooi B.S. (2021). Towards detecting red palm weevil using machine learning and fiber optic distributed acoustic sensing. Sensors.

[42] Faustov A.V., Gusarov A., Wuilpart M., Fotiadi A.A., Liokumovich L.B., Zolotovskiy I.O., Tomashuk A.L., de Schou-theete T., Megret P. (2013). Comparison of Gamma-Radiation Induced Attenuation in Al-Doped, P-Doped and Ge-Doped Fibres for Dosimetry. IEEE Trans. Nucl. Sci..

[43] Faustov A.V., Gusarov A.V., Mégret P., Wuilpart M., Zhukov A.V., Novikov S.G., Svetukhin V.V., Fotiadi A.A. (2016). Application of phosphate doped fibers for OFDR dosimetry. Results Phys..

[44] Rizzolo S., Boukenter A., Marin E., Cannas M., Perisse J., Bauer S., Mace J.-R., Ouerdane Y., Girard S. (2015). Vulnerability of OFDR-based distributed sensors to high γ-ray doses. Opt. Express.

[45] Faustov A.V., Gusarov A.V., Mégret P., Wuilpart M., Zhukov A.V., Novikov S.G., Svetukhin V.V., Fotiadi A.A. (2015). The use of optical frequency-domain reflectometry in remote distributed measurements of the γ-radiation dose. Tech. Phys. Lett..

[46] Bueno Escobedo J.L., Jason J., López-Mercado C.A., Spirin V.V., Wuilpart M., Mégret P., Korobko D.A., Zolotovskiy I.O., Fotiadi A.A. (2019). Distributed measurements of vibration frequency using phase-OTDR with a DFB laser self-stabilized through PM fiber ring cavity. Results Phys..

[47] Bueno Escobedo J.L., Spirin V.V., López-Mercado C.A., Márquez Lucero A., Mégret P., Zolotovskii I.O., Fotiadi A.A. (2017). Self-injection locking of the DFB laser through an external ring fiber cavity: Application for phase sensitive OTDR acoustic sensor. Results Phys..

[48] Muanenda Y., Oton C.J., Faralli S., Nannipieri T., Signorini A., Pasquale F.D. (2016). Hybrid distributed acoustic and temperature sensor using a commercial off-the-shelf DFB laser and direct detection. Opt. Lett..

[49] Garcia-Ruiz A., Pastor-Graells J., Martins H.F., Martin-Lopez S., Gonzalez-Herraez M. (2016). Speckle Analysis Method for Distributed Detection of Temperature Gradients With Φ OTDR. IEEE Photonics Technol. Lett..

[50] Pastor-Graells J., Martins H.F., Garcia-Ruiz A., Martin-Lopez S., Gonzalez-Herraez M. (2016). Single-shot distributed temperature and strain tracking using direct detection phase-sensitive OTDR with chirped pulses. Opt. Express.

[51] Zhang J., Zhu T., Zhou H., Huang S., Liu M., Huang W. (2016). High spatial resolution distributed fiber system for multi-parameter sensing based on modulated pulses. Opt. Express.

[52] Dang Y., Zhao Z., Tang M., Zhao C., Gan L., Fu S., Liu T., Tong W., Shum P.P., Liu D. (2017). Towards large dynamic range and ultrahigh measurement resolution in distributed fiber sensing based on multicore fiber. Opt. Express.

[53] Zhao Z., Dang Y., Tang M., Wang L., Gan L., Fu S., Yang C., Tong W., Lu C. (2018). Enabling simultaneous DAS and DTS through space-division multiplexing based on multicore fiber. J. Light. Technol..

[54] Zhang Y., Cai Y., Xiong F., Zhang M., Shan Y., Wang S., Xu W., Zabihi M., Wu J., Zhang X. (2019). A hybrid distributed optical fibre sensor for acoustic and temperature fields reconstruction. Opt. Commun..

[55] Hicke K., Eisermann R., Chruscicki S. (2019). Enhanced distributed fiber optic vibration sensing and simultaneous temperature gradient sensing using traditional C-OTDR and structured fiber with scattering dots. Sensors.

[56] Mao Y., Ashry I., Hveding F., Bukhamsin A.Y., Hong Y., Ng T.K., Ooi B.S. (2020). Simultaneous distributed acoustic and temperature sensing using a multimode fiber. IEEE J. Sel. Top. Quantum Electron..

[57] Ashry I., Mao Y., Ng T.K., Hveding F., Arsalan M., Ooi B.S. Hybrid distributed acoustic-temperature sensing using a few-mode fiber. Proceedings of the Photonic Instrumentation Engineering VII.

[58] Wang Y., Zheng H., Wu H., Huang D., Yu C., Lu C. (2024). High-sensitivity distributed temperature and salinity sensor based on frequency scanning Φ-OTDR and polyimide-coated polarization maintaining fiber. Opt. Laser Technol..

[59] Wang Y., Zheng H., Lu C. (2022). High-sensitivity distributed relative salinity sensor based on frequency-scanning φ-OTDR. Opt. Express.

[60] Huang M., Wang Z., Feng Y., Fan J., Wang Y., Lu L. (2024). Distributed vibration and temperature sensing system by multiplexed fiber scattering spectra. Appl. Opt..

[61] Bradley N., Haavik K.E., Landrø M. (2024). Estimation of Temperature Profiles using Low-Frequency Distributed Acoustic Sensing from In-Well Measurements. SPE J..

[62] Matveenko V.P., Serovaev G.S., Kosheleva N.A., Gusev G.N. (2021). On application of distributed FOS embedded into material for the mechanical state monitoring of civil structures. Procedia Struct. Integr..

[63] Belokrylov M.E., Claude D., Konstantinov Y.A., Karnaushkin P.V., Ovchinnikov K.A., Krishtop V.V., Gilev D.G., Barkov F.L., Ponomarev R.S. (2023). Method for Increasing the Signal-to-Noise Ratio of Rayleigh Back-Scattered Radiation Registered by a Frequency Domain Optical Reflectometer Using Two-Stage Erbium Amplification. Instrum. Exp. Tech..

[64] Rezak E.V., Prokopovich M.R. (2008). Accounting for the error of measuring the length of optical fiber. Bull. of PNU.

[65] Spirin V.V., Bueno Escobedo J.L., Korobko D.A., Mégret P., Fotiadi A.A. (2020). Dual-frequency laser comprising a single fiber ring cavity for self-injection locking of DFB laser diode and Brillouin lasing. Opt. Express.

[66] Spirin V.V., Bueno Escobedo J.L., Miridonov S.V., Maya Sánchez M.C., López-Mercado C.A., Korobko D.A., Zolotovskii I.O., Fotiadi A.A. (2021). Sub-kilohertz Brillouin fiber laser with stabilized self-injection locked DFB pump laser. Opt. Laser Technol..

[67] Spirin V.V., Bueno Escobedo J.L., Korobko D.A., Mégret P., Fotiadi A.A. (2020). Stabilizing DFB laser injection-locked to an external fiber-optic ring resonator. Opt. Express.

[68] Panyaev I.S., Itrin P.A., Korobko D.A., Fotiadi A.A. (2024). Sub-100-Hz DFB Laser Injection-Locked to PM Fiber Ring Cavity. J. Light. Technol..

[69] Froggatt M.E. (2009). Distributed strain and temperature discrimination in polarization maintaining fiber. U.S. Patent.

[70] Lobach I.A., Fotiadi A.A., Yatseev V.A., Konstantinov Y.A., Barkov F.L., Claude D., Kambur D.A., Belokrylov M.E., Turov A.T., Korobko D.A. (2024). Newest Methods and Approaches to Enhance the Performance of Optical Frequency-Domain Reflectometers. Sensors.

[71] Ding Z., Yang D., Du Y., Liu K., Zhou Y., Zhang R., Xu Z., Jiang J., Liu T. (2016). Distributed strain and temperature discrimination using two types of fiber in OFDR. IEEE Photonics J..

[72] Naeem K., Lee C., Linganna K., Kang C., Oh M.K., Yu N.E., Kang H., Kim B.H. (2021). Multiparameter distributed fiber sensor based on optical frequency-domain reflectometry and bandwidth-division multiplexing. IEEE Sens. J..

[73] Bao X., Li W., Qin Z., Chen L. OTDR and OFDR for distributed multi-parameter sensing. Proceedings of the Smart Sensor Phenomena, Technology, Networks, and Systems Integration.

[74] Song M., Chen G., Cui E., Yuxin Z. (2022). Multi-parameter measurement of a multi-point high-frequency vibration signal in an OFDR system. Appl. Opt..

[75] Qin Z., Qu S., Wang Z., Yang W., Li S., Liu Z., Xu Y. (2022). A fully distributed fiber optic sensor for simultaneous relative humidity and temperature measurement with polyimide-coated polarization maintaining fiber. Sens. Actuators B Chem..

[76] Mądry M., Szczupak B., Śmigielski M., Matysiak B. (2024). Simultaneous Temperature and Relative Humidity Measurement Using Machine Learning in Rayleigh-Based Optical Frequency Domain Reflectometry. Sensors.

[77] Li S., Yang X., Zhang B., Qu S., Xu Y., Liu Z., Qin Z. (2024). Potential multi-parametric OFDR system based on the wave-length-division cross-correlation method. Appl. Opt..

[78] Pedraza A., Del Río D., Bautista-Juzgado V., Fernández-López A., Sanz-Andrés Á. (2023). Study of the Feasibility of Decoupling Temperature and Strain from a ϕ-PA-OFDR over an SMF Using Neural Networks. Sensors.

[79] Zhou D.P., Li W., Chen L., Bao X. (2013). Distributed temperature and strain discrimination with stimulated Brillouin scattering and Rayleigh backscatter in an optical fiber. Sensors.

[80] Meng Y., Sui R., Liang W., Zhong H., Shan R., Xiao S., Kong Y., Fu C., Wang Y. (2024). Multicore fiber shape sensing based on optical frequency domain reflectometry parallel measurements. J. Light. Technol..

[81] Lopez-Mercado C.A., Korobko D.A., Zolotovskii I.O., Fotiadi A.A. (2021). Application of Dual-Frequency Self-Injection Locked DFB Laser for Brillouin Optical Time Domain Analysis. Sensors.

[82] Poddubrovskii N.R., Lobach I.A., Kablukov S.I. (2023). Signal Processing in Optical Frequency Domain Reflectometry Systems Based on Self-Sweeping Fiber Laser with Continuous-Wave Intensity Dynamics. Algorithms.

[83] Fotiadi A., Rafailov E., Korobko D., Megret P., Bykov A., Meglinski I. (2023). Brillouin Interaction between Two Optical Modes Selectively Excited in Weakly Guiding Multimode Optical Fibers. Sensors.

[84] Feng C., Lu X., Preussler S., Schneider T. (2019). Gain spectrum engineering in distributed brillouin fiber sensors. J. Lightw. Technol..

[85] Krivosheev A.I., Konstantinov Y.A., Barkov F.L., Pervadchuk V.P. (2021). Comparative analysis of the Brillouin frequency shift determining accuracy in extremely noised spectra by various correlation methods. Instrum. Exp. Tech..

[86] Karapanagiotis C., Krebber K. (2023). Machine Learning Approaches in Brillouin Distributed Fiber Optic Sensors. Sensors.

[87] Sheng L., Li L., Hu L., Yuan M., Lang J., Wang J., Li P., Bi Z., Yan J., Liu Z. (2020). Distributed Fiberoptic Sensor for Simultaneous Temperature and Strain Monitoring Based on Brillouin Scattering Effect in Polyimide-Coated Fibers. Int. J. Opt..

[88] Murray M.J., Murray J.B., Ogden H.M., Redding B. (2022). Dynamic temperature-strain discrimination using a hybrid distributed fiber sensor based on Brillouin and Rayleigh scattering. Opt. Express.

[89] Sabatier C., Lecoeuche V., Girard S., Mélin G., Robin T., Cadier B., Marin E. (2019). Distributed optical fiber sensor allowing temperature and strain discrimination in radiation environments. IEEE Trans. Nucl. Sci..

[90] Li A., Wang Y., Fang J., Li M.J., Kim B.Y., Shieh W. (2015). Few-mode fiber multi-parameter sensor with distributed temperature and strain discrimination. Opt. Lett..

[91] Belokrylov M.E., Konstantinov Y.A., Krivosheev A.I., Turov A.T., Stepanov K.V., Garin E.O., Pnev A.B., Fotiadi A.A. A single-scan PM-fibers polarization axes study. Proceedings of the 2022 International Conference Laser Optics (ICLO).

[92] Wang Y., Tovar P., Cotton-Dumouchel M., Chen L., Bao X. (2024). Distributed phase-matching measurement enabled dynamic temperature–strain discrimination using single chirped pulse probe. APL Photonics.

[93] Lalam N., Bukka S., Bhatta H., Buric M., Ohodnicki P., Wright R. (2024). Achieving precise multiparameter measurements with distributed optical fiber sensor using wavelength diversity and deep neural networks. Commun. Eng..

[94] Wang J., Qi B. (2025). Spatial Shape Sensing of Multi-core Optical Fiber Based on Distributed Strain Measurement and Deep Learning. IEEE Sens. J..

[95] Violakis G., Vardakis N., Zhang Z., Angelmahr M., Polygerinos P. (2025). Rapid and Accurate Shape-Sensing Method Using a Multi-Core Fiber Bragg Grating-Based Optical Fiber. Sensors.

[96] Aitkulov A., Cappelletti M., Orsuti D., Schenato L., Hayashi T., Santagiustina M., Galtarossa A., Palmieri L. (2024). Distributed twist sensing with uncoupled multi-core fibers using polarization-sensitive reflectometry. J. Light. Technol..

[97] Wang J., Gu H., Wang P., Yao G., Huang J., Liu W., Xu D., Wu S. (2025). Equivalent Self-Noise Suppression of Distributed Hydroacoustic Sensing System Using SDM Signals Based on Multi-Core Fiber. Sensors.

[98] Zhi Y., Luo P., Wang R., Qiao X. (2025). Accuracy enhancement of multi-core fiber shape sensing by a deep learning-based model with pretraining and transfer learning strategies. Opt. Lett..

[99] Feng Y., Xie W., Meng Y., Yang J., Yang Q., Ren Y., Bo T., Tan Z., Wei W., Dong Y. (2022). Multi-core fiber enabled fading noise suppression in {\phi}-OFDR based quantitative distributed vibration sensing. arXiv.

[100] Xia Q., Wang H., Zhang X., Li S., Yuan L., Yuan T. (2025). Single-channel multi-core fiber sensor for finger bending detection based on composite calibration. Opt. Express.

[101] Sherburne M., Harjes C., Klitsner B., Gigax J., Ivanov S., Schamiloglu E., Lehr J. (2024). Rapid Prototyping for Nanoparticle-Based Photonic Crystal Fiber Sensors. Sensors.

[102] Elabdein M.Z., Khedr O.E., Mohammed N.A., El-Rabaie E.S.M. (2024). Recent advances in photonic crystal fiber based chemical and industrial sensors: A review. J. Opt..

[103] Afshar M., Mehrabi M., Ferdosian Tehrani M., Mohitpour M. (2025). D-Shaped photonic crystal fiber butanol sensor affected by temperature and pressure changes. J. Opt..

[104] Hoang T.T., Pham V.D., Pham T.S., Le K.Q., Ngo Q.M. (2021). Sensitive Near-Infrared Refractive Index Sensors Based on D-Shaped Photonic Crystal Fibers. J. Nanosci. Nanotechnol..

[105] Tolen G.B., Abdykadyrov A.A., Bourdine A.V., Praporshchikov D.E., Yerishova M.O., Zaitseva E.S. (2024). Analysis of the efficiency of distributed fiber optic acoustic sensors in monitoring systems. Opt. Technol. Telecommun..

[106] Danlard I., Akowuah E.K., Opoku G. (2025). Advances in Plasmonic Photonic Crystal Fiber-Based Multiparameter Sensors: Mechanisms, Design Challenges, and Future Directions. Sens. Imaging.

[107] Li D., Li S., Huo T., Zhang Z., Gao T. (2025). Refractive Index and Temperature Dual-Parameter Sensor with Independent Channels Based on Asymmetric Dual-Core Photonic Crystal Fiber and Composite Membrane. Plasmonics.

[108] Yang F., Liu W., Mi C., Lv J., Yang L., Liu Q., Liu C. (2024). Simulation analysis of a photonic crystal fiber refractive index sensor based on a double-layer film structure and surface plasmon resonance technology. J. Opt. Soc. Am. A.

[109] Punia S., Saharia A., Ismail Y., Petruccione F., Bourdine A.V., Morozov O.G., Meshkov I.K., Singh G., Tiwari M. (2024). OAM mode propagation and supercontinuum generation in a nested photonic crystal fiber. Phys. Scr..

[110] Lin Y., Chen S.E., Wang M., Liu W. (2015). Fiber-optic fast response pH sensor in fiber Bragg gating using intelligent hydrogel coatings. Opt. Eng..

[111] Li J., Wang L., Wang X., Yang Y., Hu Z., Liu L., Huang Y. (2019). Highly conductive PVA/Ag coating by aqueous in situ reduction and its stretchable structure for strain sensor. ACS Appl. Mater. Interfaces.

[112] Pak K., Lee Y., Kim J.H., Kim M., Sim J.Y. (2025). Highly Sensitive and Robust Fiber Strain Sensor via Multiple Coating. Electron. Mater. Lett..

[113] Qu W., Chen Y., Liu S., Luo L. (2025). Advances and Prospects of Nanomaterial Coatings in Optical Fiber Sensors. Coatings.

[114] Pan’kov A.A. (2025). Statistical model for a mechanoluminescent fiber-optic sensor in a polymer indicator coating. J. Opt. Technol..

[115] Li T., Su Y., Zheng H., Chen F., Li X., Tan Y., Zhou Z. (2023). An artificial intelligence-motivated skin-like optical fiber tactile sensor. Adv. Intell. Syst..

[116] Li L., Yang F., Ma Q., Xu T., Su M., Zhao Y., Liu X. (2025). Integrating an optical fiber sensor and artificial intelligence for enhanced tactile sensing in human-computer interaction. Optica.

[117] Lin S. (2022). An ultralight, flexible, and biocompatible all-fiber motion sensor for artificial intelligence wearable electronics. Npj Flex Electron..

[118] Rana J., Sharma A.K., Prajapati Y.K. (2024). Intervention of machine learning and explainable artificial intelligence in fiber optic sensor device data for systematic and comprehensive performance-optimization. IEEE Sens. Lett..

[119] Golovastikov N.V., Kazanskiy N.L., Khonina S.N. (2025). Optical Fiber-Based Structural Health Monitoring: Advancements, Applications, and Integration with Artificial Intelligence for Civil and Urban Infrastructure. Photonics.

[120] Thévenaz L. (2025). Distributed optical fiber sensors: What is known and what is to come. Front. Sens..

[121] Pulcinelli M., D’Antoni F., Presti D.L., Schena E., Carassiti M., De Tommasi F., Merone M. (2024). Combining fiber bragg grating and artificial intelligence technologies for supporting epidural procedures. IEEE Trans. Biomed. Eng..

